# Antibody–drug conjugates for infectious and neglected tropical diseases: chemical design principles, target biology, and translational challenges

**DOI:** 10.3389/fchem.2026.1793193

**Published:** 2026-04-17

**Authors:** Desigan Reddy, Vineet Jeena, Tshephiso R. Papo, Emmanuel C. Ohaekenyem

**Affiliations:** 1 Discipline of Chemistry, School of Agriculture and Science, University of KwaZulu-Natal, Durban, South Africa; 2 Pure and Industrial Chemistry Department, Nnamdi Azikiwe University, Awka, Anambra, Nigeria

**Keywords:** antibody–drug conjugates, chemical design principles, infectious diseases, neglected tropical diseases, target biology, translational challenges, metallo-drugs

## Abstract

Antibody–drug conjugates (ADCs) are established precision treatments in oncology. Nevertheless, their application to infectious diseases and neglected tropical diseases (NTDs) is still an emerging field. In contrast to cancer cells, pathogens exhibit dynamic surface features and distinct intracellular environments, necessitating a complete redesign of the ADC architecture. This review combines chemical concepts and biological insights to outline a “pathogen-centric” framework for bacterial, viral, and parasite illnesses. We analyze target selection across various diseases, emphasizing structural accessibility and antigen stability as critical factors. A comprehensive evaluation of ADC chemical architecture is provided, focusing on linkers that respond to pathogen-specific enzymatic or environmental triggers, alongside a range of non-cytotoxic payloads, notably redox-active metallo-drugs designed to overcome antimicrobial resistance. We rigorously analyze the shift from empirical screening to AI-enhanced and structurally-informed design processes. Lastly, we look at the particular translation concerns in this field, such as the Payload Paradox and the complications that come with internalization. We discuss also sustainable biomanufacturing methods that will ensure equitable and fair access to the products. This study offers a chemistry-based framework that outlines the essential ideas required for the advancement of antibody-drug conjugates (ADCs) as targeted anti-infectives for major global infections.

## 1 Introduction

Antibody–drug conjugates (ADCs) are traditionally associated with oncology (Onc-ADCs), yet their precision targeting offers a transformative strategy for infectious diseases and neglected tropical diseases (NTDs) (Alradwan et al., 2025; Boni et al., 2020; Cen et al., 2025). The efficacy of anti-infective ADCs (ID-ADCs) is governed by the chemical interplay between antibody specificity, payload selection, and linker stability, which collectively determine the therapeutic index in complex host-pathogen environments (Alradwan et al., 2025; Lucas et al., 2021; Su and Zhang, 2021).

Unlike oncology, where Onc-ADCs target “self” cell-surface proteins, anti-infective applications (ID-ADCs) must navigate pathogen diversity, immune evasion, and inaccessible intracellular niches (Pishesha et al., 2021). Conventional therapies for bacterial infections and NTDs frequently suffer from non-specific distribution and dose-limiting toxicity; ID-ADCs address these limitations by restricting payload delivery to diseased sites (Graziani et al., 2020; Peck et al., 2019). Recent innovations in linker design, particularly lysosomal-cleavable peptide sequences, have improved circulation stability while enabling controlled, site-specific release (Balamkundu and Liu, 2023a; Gao et al., 2025; Su and Zhang, 2021).

Emerging clinical and preclinical data demonstrate that ID-ADCs can achieve meaningful efficacy against severe infections, particularly when direct antimicrobial activity is paired with immunomodulatory functions to engage host immunity (Cen et al., 2025; Shi et al., 2025). Furthermore, advances in bioconjugation have facilitated the development of peptide–drug conjugates (PDCs), which offer complementary targeting strategies with distinct manufacturing advantages for global health (Bai et al., 2019; Jadhav et al., 2025). This review evaluates the chemical principles and conjugation technologies required to unlock the full therapeutic potential of ID-ADCs as precision anti-infectives (Alradwan et al., 2025).

In this review, we refer to infectious disease–targeting antibody–drug conjugates as ID-ADCs to distinguish them from oncology ADCs.

The treatment landscape for infectious diseases and neglected tropical diseases (NTDs) has historically been dominated by small-molecule anti-infectives. Although these medicines have yielded notable improvements in clinical outcomes, their effectiveness is increasingly hindered by intrinsic pharmacological and biological constraints (Gao et al., 2018; Volpedo et al., 2019). The primary concern is the indiscriminate biodistribution of traditional antimicrobials, which may result in dose-limiting off-target toxicity, thereby impacting beneficial commensal microbiomes and causing detrimental effects including dysbiosis (Gao et al., 2018). These disturbances jeopardize patient health and exacerbate the overarching issue of antimicrobial resistance (AMR), making standard infections increasingly difficult to treat (Durbin et al., 2018).

In addition to their cytotoxic effects, many small-molecule agents exhibit insufficient penetration into critical sanctuary sites, including granulomas, the blood-brain barrier (BBB), and various intracellular compartments, where pathogens can persist and facilitate relapse (Bukowski et al., 2024; Pardridge, 2020; Wang et al., 2025a). Research demonstrates that more than 98% of small molecule drugs cannot penetrate the blood-brain barrier, significantly impairing their treatment effectiveness for central nervous system disorders (Pardridge, 2020). This is particularly alarming for NTDs, as the existing therapy pipeline is significantly inadequate, heightening the need for creative, customized ways to overcome these challenges (Castro et al., 2024; Doherty et al., 2025). Therefore, there is an urgent want for mechanistically varied therapeutic strategies, such as antibody–drug conjugates (ADCs), particularly infectious disease–targeting antibody–drug conjugates (ID-ADCs), which can provide a more targeted and effective means of combating these persistent diseases.

Recent studies have initiated investigations into novel delivery mechanisms, including nanoparticle-based systems and antibody–drug conjugates (ID-ADCs), which demonstrate potential in improving drug localization and minimizing necessary dosages (Zhang B et al., 2024; Volpedo et al., 2019; Gao et al., 2018). Nanoparticulate drug delivery systems can precisely target specific pathogens and enhance drug accessibility to sites of pathogenesis within the host, thereby yielding therapeutic outcomes with diminished side effects (Volpedo et al., 2019). These achievements emphasize a paradigm shift towards more selective and targeted therapeutics, underscoring the need for innovative therapeutic frameworks that can overcome the limitations of conventional small-molecule anti-infectives (Bukowski et al., 2024; Gao et al., 2018; Shih et al., 2024).

Antibody–drug conjugates (ADCs) are a revolutionary strategy in targeted therapy, merging the specificity of monoclonal antibodies (mAbs) with powerful payloads for the delivery of therapeutic drugs to disease locations. In oncology, antibody–drug conjugates (Onc-ADCs) have exhibited significant clinical efficacy by utilizing a strategy in which a humanized antibody specifically targets tumor-associated antigens, enabling the internalization of a cytotoxic agent, such as auristatin or maytansine, which triggers cell death (Boni et al., 2020; Schwach et al., 2022; Tonon et al., 2024). Transferring this technology to address infectious diseases necessitates a significant reassessment of the chemical structure and therapeutic objectives of ID-ADCs.

Unlike tumor cells that require elimination, the aim of developing antibody–drug conjugates for infectious diseases (ID-ADCs) is to specifically kill pathogens while minimizing harm to host cells. This inconsistency requires a thorough re-engineering of the ADC’s components. In oncology, conventional cytotoxic agents used in Onc-ADCs, which typically harm human cells, ought to be replaced in ID-ADCs with pathogen-targeted therapeutics, such as antimicrobials or antivirals (Gingrich, 2020).

Potential payloads may encompass innovative redox-active metallo-drugs or peptide conjugates engineered to elicit targeted cellular responses in pathogens (Gingrich, 2020). Moreover, linker technologies must advance to address not only human physiological characteristics but also pathogen-derived stimuli or distinctive environmental signals at infection sites, like parasite-specific proteases or modified pH levels in lysosomal compartments (Tolosa et al., 2024).

**FIGURE 1:**
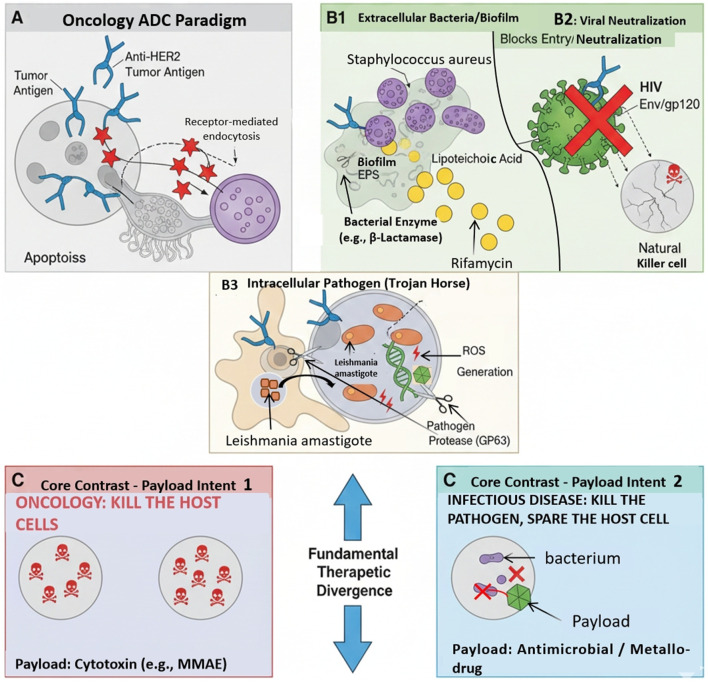
Conceptual divergence of antibody–drug conjugate designs for oncology and anti-infective applications. **(A)** Conventional oncology antibody–drug conjugates (ADCs) showing receptor-mediated binding to tumor-associated antigens (e.g., HER2), followed by endocytosis and intracellular release of cytotoxic payloads such as monomethyl auristatin E (MMAE), resulting in host-cell apoptosis. **(B)** Anti-infective ADC strategies illustrating alternative targeting and payload deployment modes: **(B1)** extracellular targeting of conserved bacterial surface components or biofilm matrices to enable localized antimicrobial release; **(B2)** viral targeting through envelope glycoprotein recognition, leading to viral neutralization and immune-mediated clearance of infected cells; and **(B3)** intracellular delivery into infected host cells, where pathogen-specific enzymatic activity or acidic compartments trigger payload release within the pathogen niche. **(C)** Comparative representation of payload intent, highlighting host-cell elimination in oncology ADCs versus pathogen-selective eradication in anti-infective ADCs. Abbreviations: ADC, antibody–drug conjugate; EPS, exopolysaccharides; HER2, human epidermal growth factor receptor 2; MMAE, monomethyl auristatin E; ROS, reactive oxygen species.

This essential discrepancy in treatment goals requires a comprehensive reconfiguration of the ADC’s components, a conceptual transformation depicted in Figure 1.

Consequently, the design of antibody–drug conjugates for infectious disorders (ID-ADCs) necessitates novel thought rooted in the principles of chemical biology, structural microbiology, and pathogenesis. A multidisciplinary strategy is essential for effective ID-ADC development, integrating medicinal chemistry and bioinorganic techniques to create stable conjugations and build pathogen-responsive linkers (Kwon, 2021). Simultaneously, progress in structural biology allows researchers to delineate epitopes on pathogen surfaces, discern antigenic variations, and create novel payloads specifically for ID-ADCs. In this context, the disease biology influencing infection environments—factors such as pH, redox state, and protease activity—must be meticulously considered to guide linker activation and payload release (Zhu K et al., 2023; Tolosa et al., 2024).

Effective infectious disease–targeting antibody–drug conjugates (ID-ADCs) require complex integration across multiple scientific disciplines, reflecting design considerations that differ substantially from conventional oncology ADCs. The significance of medicinal and bioinorganic chemistry in developing stable conjugations and engineering pathogen-responsive linkers is paramount, particularly as these modifications directly influence release kinetics, payload activation, and target specificity (Kwon, 2021). Unlike oncology ADCs, which predominantly rely on lysosomal processing following internalization into tumor cells, ID-ADCs may require extracellular or pathogen-triggered release mechanisms tailored to infection-specific biological cues.

Techniques in structural biology, such as cryo-electron microscopy and computational modeling platforms (e.g., AlphaFold), are essential for identifying accessible and conserved pathogen epitopes while elucidating their structural dynamics (Zhu K et al., 2023). These structural insights are particularly critical in infectious diseases, where antigen variability and resistance mechanisms necessitate precise epitope selection. The findings from such investigations aid in identifying optimal antigens and improving therapeutic selectivity.

Furthermore, a comprehensive understanding of pathogen biology requires consideration of the distinctive characteristics of the infection microenvironment, including localized pH shifts, enzymatic activity, biofilm formation, and the presence of reactive species, all of which directly influence ID-ADC stability and efficacy (Kwon, 2021). The deliberate integration of chemistry, structural biology, and pathogen biology therefore advances ID-ADCs from theoretical adaptations of oncology platforms to purpose-built antimicrobial therapeutics, significantly enhancing their translational potential in combating infectious diseases.

This thorough study seeks to examine the domain of antibody–drug conjugates designed for infectious and neglected tropical diseases (ID-ADCs), consolidating material from recent years, namely from 2018 to 2025. This period signifies a transition from theoretical proposals to focused preclinical investigation of ID-ADCs. Although fundamental oncology literature is cited for necessary context, our emphasis is on recent advancements crucial for delineating the present technological landscape and prospective trajectories (Gingrich, 2020).

This review’s key components involve evaluating the molecular architecture of ID-ADCs, emphasizing the distinctive design constraints dictated by pathogen biology, and differentiating them from Onc-ADCs. We will assess preclinical initiatives targeting bacterial, viral, and parasitic infections, highlighting not only achievements but also significant chemical and biological differences from conventional cancer treatments. Particular emphasis is placed on advancements in linker technology and the investigation of innovative non-cytotoxic payloads, such as metallo-drug complexes with distinct biochemical processes (Gingrich, 2020; Labant, 2024). Finally, a contemporary computational framework for the design of ID-ADCs will be introduced, accompanied by an assessment of sophisticated *in vitro* and *in vivo* models essential for predictive testing.

This review seeks to synthesize insights from structural biology, medicinal chemistry, pathogenesis, and translational pharmacology to identify critical knowledge gaps and propose novel, integrated solutions for advancing ID-ADCs into clinical applications for some of the most resilient infectious diseases globally.

While Onc-ADCs are well established in oncology, their application to infectious diseases and neglected tropical diseases (NTDs) remains insufficiently synthesized. Current literature predominantly elucidates linker chemistries, cytotoxic payloads, and pharmacokinetics tailored to tumor biology (Hafeez et al., 2020; Long et al., 2025). Existing research into ID-ADCs often attempts to mirror oncology frameworks without addressing the unique biological hurdles of infection (García-Alonso et al., 2018; Yu et al., 2022).

This review addresses this knowledge deficiency by integrating pathogen biology, inorganic chemistry, and translational pharmacology. It provides a prospective analysis across four unique pillars:

1. Pathogen-Centric Chemical Design: We analyze how the distinct biology of bacteria, viruses, and parasites—including surface antigen profiles and intracellular environments—dictates the design of specialized antibodies, linkers, and payloads for ID-ADCs.
2. Structural Biology as a Design Engine: We underscore the role of cryo-electron microscopy and computational tools (e.g., AlphaFold) in epitope mapping and the rational design of pathogen-responsive components for ID-ADCs (Chaundler et al., 2023; Zimmerman and Esteva, 2024).
3. Beyond Cytotoxicity: Metallo-Drug Payloads: We emphasize non-cytotoxic payloads, specifically bioinorganic complexes like tetraaza macrocycles and phenanthroline systems, as therapeutic strategies in ID-ADCs to circumvent traditional resistance (Walker et al., 2019).
4. Integrated Translational Roadmap: We unify advanced models (organ-on-chip, 3D granulomas), AI-driven frameworks, and regulatory strategies into a cohesive development pipeline for ID-ADCs (Hafeez et al., 2020; Yu et al., 2022).

By synthesizing these fields, this study offers a specialized framework to transition ID-ADCs from conceptual design to clinical candidates for the world’s most challenging infections (García-Alonso et al., 2018; Tsuchikama et al., 2024).

## 2 Molecular architecture of anti-infective ADCs (ID-ADCs)

Conventional oncology ADCs are designed around three core principles: (i) antibodies targeting tumor-associated antigens with high internalization rates, (ii) linkers cleaved under lysosomal conditions, and (iii) highly potent cytotoxic payloads that induce apoptosis in rapidly dividing host cells (Chen et al., 2025). These principles evolved in the relatively homogeneous and host-derived environment of solid tumors. In contrast, infectious diseases present heterogeneous, extracellular or intracellular pathogens with distinct biochemical microenvironments. Consequently, the molecular architecture of ID-ADCs must be fundamentally reconfigured at each structural level—antibody, linker, and payload—to account for the dynamic pathogen environment (Khongorzul et al., 2020).

### 2.1 Antibody engineering: recognition and penetration

Antibodies for infectious diseases (ID-ADCs) must balance high specificity with optimal tissue kinetics. Variable domains (Fab) target epitopes uniquely associated with the pathogen or infected host cell, prioritized by epitope conservation to prevent resistance through antigenic drift (Motley et al., 2019). Binding affinity requires precise calibration; excessively high affinity can impede deep tissue penetration or trigger sequestration on extracellular pathogens, particularly in high-burden chronic infections (Ye et al., 2020).

The Fc region provides secondary therapeutic value via antibody-dependent cellular cytotoxicity (ADCC) (Su et al., 2021). Furthermore, modifying Fc-neonatal receptor (FcRn) affinity can extend serum half-life—a benefit for chronic tuberculosis management—though rapid clearance may be preferred in acute sepsis scenarios (Lovey et al., 2021). Unlike oncology, where antigen overexpression drives targeting, ID-ADCs must prioritize epitope conservation, accessibility across pathogen life stages, and avoidance of antigenic variation.

### 2.2 Linker chemistry: pathogen-responsive triggers

In contrast to the host-centric lysosomal triggers (low pH, cathepsins) used in oncology (Onc-ADCs), ID-ADCs increasingly utilize “biosensing” linkers cleaved by pathogen-specific fingerprints. These include pathogen-derived proteases such as Leishmania GP63 or malaria falcipains, or the localized reactive oxidative stress (ROS) characteristic of bacterial inflammatory niches (O’Leary et al., 2023; Ruddle et al., 2019; Sasso et al., 2023). This transition ensures the “molecular fuse” remains stable in systemic circulation while facilitating rapid release only upon interaction with the targeted pathogen environment (Barros et al., 2021). Thus, ID-ADCs require pathogen-responsive rather than host-lysosomal activation logic.

### 2.3 Payload design: beyond cytotoxicity

The payload objective shifts from inducing apoptosis in host cells (Onc-ADCs) to selective pathogen lethality (ID-ADCs) (Riaz et al., 2025). While traditional cytotoxins like auristatins remain useful for eradicating infected reservoirs, the emphasis is moving toward:

1. Targeted Antimicrobials: High-concentration delivery of antibiotics, antivirals, or antiparasitics (ID-ADCs) (Peukert et al., 2023).
2. Alternative Modalities: Immunomodulators (e.g., TLR agonists) and metallo-drug complexes that utilize redox-active mechanisms to bypass traditional resistance (ID-ADCs) (Mookherjee et al., 2020; Wu et al., 2022).

This represents a paradigm shift from host-cell cytotoxicity to pathogen-selective lethality.

### 2.4 Site-specific conjugation

To avoid the heterogeneous drug-to-antibody ratios (DAR) of stochastic lysine/cysteine conjugation, precision engineering is essential for both Onc-ADCs and ID-ADCs (Benjamin et al., 2019; Falck and Müller, 2018). Techniques utilizing modified cysteines, unnatural amino acids (e.g., p-acetylphenylalanine), and enzymatic labels (Sortase, Transglutaminase) allow for defined DAR and strategic payload positioning away from antigen-binding sites (Adhikari and Chen, 2025; Falck and Müller, 2018). Bioorthogonal copper-free click chemistry further enables the stable attachment of sensitive, non-traditional payloads (Galkin et al., 2018).

### 2.5 Structural biology and computational design

Structural biology provides the atomic-resolution framework necessary for rational ID-ADC design. Cryo-EM and X-ray crystallography delineate targetable epitopes and clarify antibody-antigen binding modes (Pal et al., 2018; Zhu K et al., 2023). These are augmented by AlphaFold2/3, which predicts structures for antigens lacking experimental data, and Molecular Dynamics (MD), which simulates linker flexibility and susceptibility to premature cleavage within specific enzymatic pockets (Ruma et al., 2025; Schoehn et al., 2023). For example, structural and computational analyses of the Leishmania surface metalloprotease GP63 have identified conserved extracellular domains suitable for antibody targeting, while docking and MD simulations have characterized accessibility and stability within parasite-specific proteolytic niches, illustrating how structural mapping can directly inform epitope selection and activation logic in ID-ADC development (Ali et al., 2025). This integrated computational-experimental pipeline transforms ADC design from empirical trial-and-error into a systematic engineering science (Gonen, 2025; Mitra, 2019).

## 3 Structural biology of antigen selection in infectious diseases

The initial phase in the creation of antibody-drug conjugates (ID-ADCs), which involves the selection of an appropriate target antigen, is considerably more intricate than for Onc-ADCs. In cancer, targets are generally overexpressed self-proteins characterized by very stable structures. Conversely, pathogen targets may display significant diversity, are alien to the host organism, and are presented within the dynamic framework of host-pathogen interactions.

Thus, structural biology is not simply an auxiliary instrument in this process; it is crucial for transforming antigen selection into a logical, physics-oriented design challenge. The distinct obstacles differ significantly among pathogen groups, as comprehensively outlined in Table 1, which assesses the molecular ‘locks’ that delineate the current Frontier of anti-infective (ID-ADC) research.

**TABLE 1:** Comparative antigen biology and targeting challenges across pathogen classes.

Pathogen class	Representative pathogens	Ideal antigen characteristics	Exemplary target candidates	Primary targeting and internalization challenge	Key structural biology insight needed
Bacteria (Extracellular)	*Staphylococcus aureus*, *Pseudomonas aeruginosa*	Conserved surface polymers; high copy number; exposed epitope	Lipoteichoic acid (LTA), Psl exopolysaccharide, capsular polysaccharides	Non-internalizing targets; antibodies may opsonize but not guarantee payload delivery into bacterial cytosol. Dense cell wall barrier	Cryo-electron tomography (Cryo-ET) mapping of capsule/biofilm matrix architecture; epitope accessibility on rigid polysaccharides (Matveev et al., 2019)
Bacteria (intracellular/facultative)	*Mycobacterium tuberculosis*, *Listeria* spp.	Host cell surface marker of infection; pathogen antigen exposed during trafficking	Host macrophage scavenger receptors; mycobacterial arabinogalactan components	Misdirected trafficking: ADC must reach the specific pathogen-containing vacuole (e.g., phagosome) and not be recycled/degraded	Structural definition of host receptor changes upon infection; dynamics of phagosomal membrane proteins (Wang Y et al., 2024)
Viruses	HIV, influenza, RSV, HBV	Conserved viral envelope glycoprotein; host receptor induced on infected cell	HIV Env gp120/gp41, Influenza HA stem, RSV F protein, infected cell markers (e.g., CD4-HIV complex)	Antigenic variation and low density: Hypervariable loops shield conserved epitopes; low antigen density on latently infected cells	Cryo-electron microscopy (Cryo-EM) of native envelope trimers to identify conserved, conformational epitopes; dynamics of viral fusion intermediates (Yuan et al., 2019)
Protozoan Parasites (Blood/Tissue)	Plasmodium spp. (malaria), Leishmania spp., Trypanosoma spp.	Stage-specific, abundant surface protein or glycoconjugate; invariant functional domain	P. falciparum PfEMP1 (conserved subdomain), Leishmania GP63/LPG, Trypanosoma VSG (invariant region)	Extreme antigenic variation and lifecycle complexity: Dense glycocalyx (VSG coat); rapid switching (PfEMP1); intracellular niche (Leishmania)	Structural mapping of invariant protein folds; protease active site architecture (GP63) (Amaral et al., 2019; Pericolini, 2018)
Fungi	*Candida* albicans, Aspergillus fumigatus	Cell wall polysaccharides (β-glucans, chitin) or specific surface proteins	β-(1,3)-D-glucan, mannoproteins, melanin	Rigid, complex cell wall that masks protein antigens; immune evasion via molecular mimicry	Structural analysis of cell wall assembly and surface protein presentation under host conditions; dynamic changes in cell wall composition during infection (Fujita et al., 2019; Gautam et al., 2024; Tateishi et al., 2025)

### 3.1 Structural requirements of an ideal pathogen antigen

To qualify for ID-ADC development, an antigen must meet a rigorous and comprehensive array of biophysical and biological parameters, all of which can be evaluated by structural techniques. Surface accessibility and epitope exposure are essential; the target antigen must be solvent-exposed and not obstructed by glycans, adjacent proteins, or densely arranged surface structures. Methods such as cryo-electron tomography (cryo-ET) can proficiently delineate the “antigenic landscape” of pathogens, facilitating the distinction between concealed domains and accessible surfaces (Grant et al., 2020; Kwon et al., 2018).

Furthermore, a high copy number and dense clustering of antigens are frequently essential for promoting effective ID-ADC binding and internalization. These properties can be measured using techniques such as quantitative fluorescence microscopy and single-particle analysis (Wintjens et al., 2020). Moreover, conservation among strains and throughout the pathogen’s life cycle is crucial to avert therapeutic evasion; hence, comparative structural bioinformatics can be utilized to pinpoint invariant areas within otherwise changeable proteins (Ghorbani et al., 2020).

Moreover, a crucial characteristic of the chosen antigen is its capacity for internalization; the antibody-antigen combination must be endocytosed effectively. This requirement frequently necessitates that the antigen be integrated into a native endocytic pathway. Studies on structural dynamics can elucidate whether antibody binding prompts conformational alterations conducive to internalization (Wang J et al., 2024).

### 3.2 Cryo-EM and crystallographic mapping of pathogen epitopes

High-resolution structural methods are transforming the identification and characterization of targetable epitopes for ID-ADCs. Cryo-electron microscopy (cryo-EM), especially in the context of Fab-bound pathogen surface complexes, has become an essential technique for *de novo* epitope mapping. Cryo-EM is particularly adept at identifying conformational epitopes, which consist of residues assembled by protein folding and are frequently undetected by linear peptide assays. This property is crucial for targeting intricate viral glycoproteins, including the HIV Envelope trimer and the SARS-CoV-2 Spike protein, as well as the variable surface glycoprotein (VSG) layers present in trypanosomes (Derking and Sanders, 2021; Li et al., 2020).

X-ray crystallography of antigen-Fab complexes offers atomic-level insights into the specific chemical interactions, including hydrogen bonds and salt bridges, that characterize the paratope-epitope interface. This precise knowledge is essential for elucidating the structural basis of antibody neutralization and provides a platform for the engineering of affinity-matured antibody variants (Paull et al., 2019).

### 3.3 Conformational masking and antigenic variation

Pathogens often utilize structural dynamics as a principal mechanism for immune evasion, presenting significant obstacles for ID-ADC targeting. Conformational masking is a strategy in which epitopes are temporarily concealed. An exemplary case is the influenza hemagglutinin (HA), which experiences significant conformational alterations during membrane fusion; certain conserved epitopes are exclusively revealed in the post-fusion state (Das et al., 2018; Mitran et al., 2019). Likewise, specific viral proteins assume “closed” conformations that obscure conserved areas from immune detection (Lindesmith et al., 2018). This underscores the need for novel targeting strategies in the development of ID-ADCs against infectious pathogens.

Furthermore, antigenic variation, as demonstrated by the PfEMP1 protein in malaria, entails the genetic control of surface protein expression. This unpredictability hinders the identification of stable target epitopes (Ras-Carmona et al., 2021). Structural investigations of various variations can uncover conserved subdomains or pivotal structural “linchpins” crucial for protein function and stability, hence offering possible targets for ID-ADCs. Methods like time-resolved structural biology and the examination of antigen-antibody complexes under diverse settings are essential for addressing these evasion strategies (Mitran et al., 2019; Rao et al., 2023).

### 3.4 Identification of ADC-compatible epitopes using structural bioinformatics

In the absence of experimental structures—frequently encountered with viruses causing neglected tropical diseases—computational predictions are crucial. AlphaFold2 and RoseTTAFold are capable of producing high-confidence three-dimensional models for a wide array of pathogen proteins, offering an essential preliminary assessment of overall folding and surface topology (Turner et al., 2018). Consequently, *in silico* epitope scanning techniques can forecast immunogenic regions; nonetheless, for ID-ADC creation, the analytical emphasis must evolve.

In this context, the focus transitions from general immunogenicity to structural appropriateness, preferring rigid, well-ordered loops over flexible linkers. Evaluating solvent accessibility and the closeness of putative epitopes to the membrane is essential for identifying possible internalization signals (Inoue et al., 2022). Molecular dynamics (MD) simulations enhance this approach by assessing the flexibility and conformational stability of anticipated epitopes over time. A rigid, stable epitope is favored for reliable and high-affinity ID-ADC binding, whereas a highly flexible loop may lead to poor binding kinetics and reduced selectivity (Guthmiller et al., 2021).

### 3.5 Case in point: the contrast with oncology targets

This systematic, structured methodology highlights the essential distinction in antigen selection between viral illnesses and cancer. A typical cancer target (Onc-ADC), like HER2, is a unique, stable human protein distinguished by a precise structure and persistent overexpression in tumor cells. Conversely, an ID-ADC target for Plasmodium falciparum may pertain to a particular conformational state of the hypervariable antigen PfEMP1, which is only produced on the membrane of infected red blood cells during the blood stage of infection. For *Mycobacterium tuberculosis*, the target may be a complex arabinogalactan structure within the resilient cell wall, requiring antibodies that identify specific polysaccharide patterns. This presents a uniquely distinctive structural barrier compared to conventional protein-epitope recognition commonly observed in oncology (Guo et al., 2018; Tas et al., 2022).

Consequently, the antigen selection phase for ID-ADCs transcends a mere screening process; it necessitates an extensive structural and biophysical analysis of the pathogen-host interface. This procedure necessitates a cohesive pipeline that amalgamates high-resolution imaging, computational forecasting, and dynamic simulation to discern rare epitopes that meet both biological significance and chemical feasibility (Ferdous and Martin, 2018; Zhai et al., 2022).

## 4 Current advancements of ADCs in infectious diseases

The translational landscape for ID-ADCs is marked by innovative proof-of-concept studies that collectively affirm the therapeutic potential of this strategy while exposing considerable hurdles specific to its implementation. Advancements differ significantly among pathogen categories, affected by factors like antigen accessibility, the presence of suitable *in vivo* models, and the degree to which disease biology conforms to or deviates from recognized Onc-ADC frameworks. This section examines significant advancements in bacterial, viral, and parasite diseases, evaluated through the combined lenses of chemistry, structural biology, and pathogen-specific biology. Table 2 delineates the translational environment, demonstrating the application of customized antibody formats and pathogen-responsive linkers to address the shortcomings of traditional therapies, emphasizing both encouraging proofs-of-concept and significant disparities with cancer medicines.

**TABLE 2:** Landscape of preclinical antibody–drug conjugates for infectious diseases highlighting target biology, linker strategies, and payload classes.

Pathogen/Disease	Target antigen	ADC format/platform	Payload class and agent	Linker chemistry and cleavage trigger	Development stage and key insight (2025 update)	Primary References
Bacterial						
Staphylococcus aureus (MRSA)	Lipoteichoic Acid (LTA)/Wall Teichoic Acid (WTA)	THIOMAB™ (Genentech)/IgG	Antibiotic: Rifamycin-class derivative	Valine-Citrulline (Val-Cit) – Cathepsin B cleavable	Phase 1b (Completed). Proof-of-concept for “Trojan horse” strategy. Demonstrates ∼100x potency over free drug in models	Dannheim et al. (2022), Matveev et al. (2019), Peck et al. (2019), Usama et al. (2021)
Pseudomonas aeruginosa	Psl exopolysaccharide/O-antigen (LPS)	Human IgG1/Site-specific (Click)	Antibiotic: Ciprofloxacin/Bacteriocin: Pyocin S5	Non-cleavable (thioether)/Siderophore-mimicry	Preclinical. Superior biofilm eradication vs. free drug. Demonstrates penetration of Gram-negative outer membrane	Wang et al. (2024), Wang et al. (2025b), Guo et al. (2023)
Mycobacterium tuberculosis	(Conceptual: mycolic acid/host phagosome marker)	(Conceptual ADC/Fragment)	Antibiotic: Bedaquiline derivative/Metallo-drug: Redox-active complex	pH-sensitive (phagosomal)/ROS-sensitive (Boronate)	Preclinical (Conceptual/Exploratory). Aims to target granuloma hypoxia/oxidative burst. Key challenge is validated, internalizing antigen	Braniewska et al. (2024), Tashima (2022)
Viral						
Influenza A and B	Neuraminidase (NA)	Drug-Fc Conjugate (DFC) – CD388 (Cidara)	Antiviral: Zanamivir-based inhibitor	Non-cleavable	Phase 3. Leading candidate for universal seasonal prophylaxis. Shows efficacy against H5N1 and resistant strains	Döhrmann et al. (2024), Lee et al. (2019)
HIV-1	Envelope gp120/Host CD4 (latent reservoir)	Immunotoxin (IgG-PE)/ADC (e.g., VRC01-ADC)	Protein Toxin: *Pseudomonas* Exotoxin A (PE38)/Cytotoxin: MMAE	Furin-cleavable (within toxin)/Cathepsin-B cleavable	Early Clinical/Preclinical. “Shock and kill” strategy for latent reservoir. High immunogenicity is a hurdle for toxin-based approaches	Bossowska-Nowicka et al. (2019), Pericolini (2018), Wang S et al. (2022), Vafadar et al. (2020)
Respiratory Syncytial Virus (RSV)	Fusion (F) protein (Palivizumab epitope)	Palivizumab (humanized mAb) conjugate	Cytotoxin: Duocarmycin (DNA alkylator)	Protease-cleavable (Val-Cit)	Preclinical. Significant viral load reduction in cotton rat model vs. naked mAb. Highlights safety concerns of cytotoxic payloads in pediatrics	Amaral et al. (2019), Stoessel et al. (2020), Tarasova et al. (2020)
Parasitic						
Leishmania spp. (Visceral)	Surface protease GP63/Lipophosphoglycan (LPG)	IgG/scFv constructs	Antiparasitic: Amphotericin B (AmB)/Metallo-drug: Cu-Cyclam complex	Pathogen-Specific: GP63-cleavable peptide linker	Preclinical. Landmark proof-of-concept for pathogen-activated linker. Metallo-drug iteration explores catalytic ROS generation	Sasso et al. (2023), Fujita et al. (2019), Jiang X et al. (2024), Wang X et al. (2022)
Plasmodium falciparum (Malaria)	Infected RBC antigen (e.g., PfEMP1 variant)	Anti-PfEMP1 mAb	Antiparasitic: Artemisinin derivative/Metallo-drug: Ferroquine-Ru hybrid	Not specified/Designed for intra-parasitic activation	Preclinical *in vitro*. Selective killing of infected RBCs. Major challenge is PfEMP1 antigenic variation; requires targeting conserved subdomains	Cho et al. (2022), Tateishi et al. (2025), Torres et al. (2022), Wang et al. (2020)
Trypanosoma spp.	(Exploratory)	(Conceptual)	Metallo-drug: Ru/Ag complexes (e.g., Ru-Clotrimazole)	(Conceptual)	Preclinical (Early). Metal coordination enhances activity of known antiparasitic ligands	Hafeez et al. (2020), Qian et al. (2023)
Strategic category overview						
Oncology Benchmark	TROP-2, HER2, etc.	Site-specific, high DAR (e.g., Dato-DXd)	Cytotoxin: Topoisomerase I inhibitor (Deruxtecan)	Tetrapeptide-based (GGFG)	Approved. Mature paradigm: targeting overexpression for internalization and lysosomal release	Hafeez et al. (2020), Shim (2020)
Anti-infective Strategic Pivot	Pathogen surface polymers, conserved glycoproteins	DFC, THIOMAB, pathogen-responsive designs	Diverse: Antimicrobials, Immunomodulators, Metallo-drugs	Pathogen-specific (protease/pH/ROS)	Preclinical to Phase 3. Shift from killing host cells to eliminating pathogens with precision	Grunst et al. (2023), Shivatare et al. (2023)

### 4.1 Bacterial antibody-drug conjugates: addressing resistance and intracellular environments

The advancement of bacterial ID-ADCs is chiefly motivated by two objectives: addressing pervasive antimicrobial resistance (AMR) and facilitating the delivery of active medicines into difficult-to-access intracellular compartments. Advanced instances illustrate a “Trojan horse” technique; for example, ID-ADCs targeting *Staphylococcus aureus* employ antibodies against the conserved cell wall component lipoteichoic acid (LTA), coupled to a rifamycin analogue. This architecture allows the ID-ADC to be ingested by infected phagocytes, immediately delivering the antibiotic payload to the intracellular milieu of methicillin-resistant *S. aureus* (MRSA) (Qin et al., 2023; Travassos et al., 2018). An ID-ADC aimed at the Psl exopolysaccharide of *Pseudomonas aeruginosa*, linked to ciprofloxacin, has shown improved effectiveness against biofilms *in vivo*, where conventional antibiotics are ineffective (Lavender et al., 2025).

These examples underscore a significant transition towards focusing on conserved, non-protein surface components (such as polysaccharides or teichoic acids), a choice influenced by the structural biology of bacterial cell envelopes. Nevertheless, appropriate and conserved protein targets that consistently enable internalization are predominantly unrecognized for numerous priority AMR pathogens, including *Acinetobacter* baumannii and carbapenem-resistant Enterobacteriaceae, highlighting a substantial knowledge deficiency in the domain (Lovey et al., 2021).

### 4.2 Viral ID-ADCs: pursuing the infected cell

In the domain of viral ID-ADCs, techniques often adopt a “cell suicide” methodology akin to Onc-ADC principles, with the objective of eradicating virus-producing or latently infected host cells. Initial endeavors with HIV entailed conjugating antibodies that target gp120 or host CD4 to protein toxins such as *pseudomonas* exotoxin A (PE). Despite the efficacy of these immunotoxins in eradicating HIV-infected cell lines *in vitro*, they encounter significant translational obstacles, such as the immunogenicity of bacterial toxin payloads and challenges in penetrating and targeting the latent viral reservoir within anatomical sanctuaries (Muppa et al., 2024; Zelter et al., 2022).

An advanced candidate is an ID-ADC targeting Respiratory Syncytial Virus (RSV), whereby the monoclonal antibody palivizumab is linked to the DNA-alkylating cytotoxin duocarmycin. This construct has demonstrated enhanced viral reduction relative to the unmodified antibody in animal studies. Nonetheless, its dependence on a traditional Onc-ADC payload reveals a fundamental conflict: although it demonstrates effective cytotoxicity against infected cells, the associated risks of host cell toxicity may be tolerable for severe RSV cases in high-risk infants but problematic for wider therapeutic or prophylactic applications (Ahmad, 2022; Cavaco et al., 2022).

### 4.3 Parasitic ID-ADCs: innovative pathogen-responsive chemistry

Significant advancements in parasitic ID-ADCs are being made, especially concerning intracellular parasites, where linker chemistry can be meticulously customized to align with pathogen biology. An exemplary case is the ID-ADC for visceral leishmaniasis, which connects antibodies targeting the surface protease GP63 or lipophosphoglycan (LPG) to amphotericin B through a GP63-cleavable peptide linker. This method illustrates a sensible, pathogen-informed design: the parasite’s plentiful surface protease acts as the catalyst for payload release, guaranteeing activation mostly at the site of infection (Saeed et al., 2023; Ye et al., 2020). Proof-of-concept studies for malaria have shown that antibodies directed against variant surface antigens on infected red blood cells (iRBCs), when conjugated to artemisinin derivatives, selectively eliminate iRBCs *in vitro*. The challenge resides in antigenic variation; the principal target, PfEMP1, demonstrates significant diversity, requiring either the identification of conserved subdomains via computational methods or the formulation of ID-ADC cocktails, thereby complicating both development and manufacturing (Mayer and Impens, 2021; Torres et al., 2022).

### 4.4 Insights from structural biology regarding variations with Onc-ADCs

An examination of these case studies uncovers essential operational distinctions between Onc-ADCs and ID-ADCs. The characteristics of the antigen vary significantly: oncology targets are often “self” proteins with stable structures, whereas infectious illness targets may consist of alien non-protein polymers or highly changeable proteins. Secondly, the purpose of the payload differs fundamentally; in oncology, it targets the host cell’s machinery, whereas in infectious diseases, it must directly interact with the pathogen or disrupt the host-pathogen interaction, frequently resulting in pharmacological discrepancies in cytotoxic effects (Abdalhamed et al., 2021; Mugenyi, 2025).

The rationale for the linker design is fundamentally redefined. Onc-ADC linkers utilize universal host-cell pathways, such as lysosomal proteases and low pH, whereas the most promising linkers for ID-ADCs, including GP63-cleavable variations, are engineered to be triggered by pathogen-specific enzymes (Liao et al., 2021). This design approach requires an in-depth comprehension of pathogen biochemistry and signifies a significant shift from conventional oncology paradigms. Although the existing preclinical portfolio is constrained, it underscores an essential principle: success necessitates not only adherence to the oncology framework but also a reorientation towards structurally characterized pathogen epitopes, pathogen-activated linker chemistries, and targeted payloads directed at infectious agents (Бурмистрoв et al., 2021).

### 4.5 The current clinical landscape: a comparative analysis of Onc-ADCs and ID-ADCs

By late 2025, the antibody-drug conjugate (ADC) sector exhibits a significant imbalance in development between oncology and infectious illnesses. Although cancer has entered a prosperous “Golden Age,” evidenced by 15 FDA-approved Onc-ADCs and more than 200 candidates in clinical development, the application for infectious and neglected tropical diseases (NTDs) is still in a revolutionary preclinical and early clinical phase. The “Translational Gap” arises not from technology shortcomings, but from the heightened stability and safety standards required in the non-oncological context.

#### 4.5.1 The oncology benchmark: commercial maturity

Between 2018 and 2025, oncology witnessed the approval of numerous significant medications, including Trastuzumab deruxtecan (Enhertu) and Sacituzumab govitecan (Trodelvy). These “Third-Generation” Onc-ADCs employ site-specific conjugation and powerful Topoisomerase I inhibitor payloads. The recent introductions in 2025—Datopotamab deruxtecan and Telisotuzumab vedotin—enhance the progression towards more sophisticated linker chemistries and varied targets such as TROP-2 and c-Met. This established success offers a structural and regulatory framework that infectious disease experts are beginning to adopt.

#### 4.5.2 The ID-ADC landscape: emerging pathogen-centric strategies

Although there is no FDA-approved ID-ADC, the period from 2021 to 2025 has signified a crucial strategic shift. The discipline has transitioned from simply replicating oncology paradigms to adopting pathogen-centric methodologies, emphasizing pathogen-responsive linkers, novel non-cytotoxic payloads, and target antigens validated through structural biology. This evolution sets the stage for the next-generation of anti-infective therapeutics, informed by lessons learned from Onc-ADCs while addressing the distinct pharmacological and safety requirements of infectious disease applications.

1. Significant Advancement in Viral Prophylaxis: The foremost development is CD388 (Cidara Therapeutics), a Drug-Fc Conjugate (DFC) now undergoing Phase 3 studies for universal influenza prevention. In 2025, CD388 reached a significant milestone by finalizing target enrollment for its pivotal ANCHOR study, indicating that the inaugural approval for a non-oncology bioconjugate is forthcoming. This represents a landmark achievement for ID-ADC development in viral diseases.
2. DSTA4637S (Genentech): Continues to be the structural innovator for targeting bacterial reservoirs in diseases. Utilizing a THIOMAB antibody platform to target wall teichoic acid on *S. aureus*, this ID-ADC effectively eradicates intracellular bacterial reservoirs that evade conventional antibiotics, demonstrating the value of pathogen-responsive design principles.
3. The NTD and HIV Frontier: Recently (2024–2025), focus has shifted toward chronic and neglected diseases. The ID-ADC VRC01-ADC is under investigation for HIV “Shock and Kill” tactics, while novel preclinical candidates for leishmaniasis are integrating metallo-drug payloads (e.g., Cu-Cyclam complexes) to generate catalytic reactive oxygen species and circumvent conventional resistance mechanisms. These developments underscore the emerging pipeline of ID-ADCs informed by structural biology and pathogen-centric chemistry.

**FIGURE 2:**
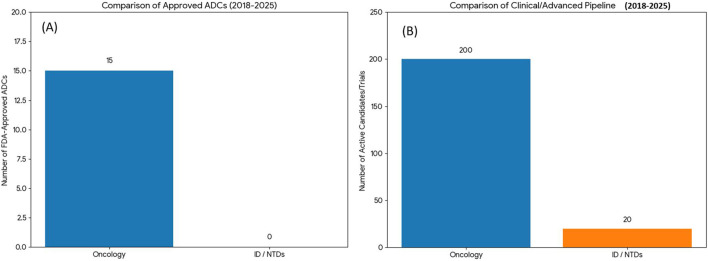
Comparative Analysis of the ADC Global Landscape (2018–2025). **(A)** Cumulative Regulatory Approvals: Temporal tracking of FDA-authorized antibody-drug conjugates (Onc-ADCs) from 2018 to 2025. Data highlights the maturity of oncology-based therapies (n ≈ 15–20) relative to the current absence of clinical approvals for infectious disease-targeting ADCs (ID-ADCs) or neglected tropical disease (NTD) indications. **(B)** Global Clinical and Advanced Pipeline (2025): Distribution of active ADC candidates currently in clinical trials or advanced development. The disparity between oncology (∼200 Onc-ADC candidates) and ID/NTDs (∼20 ID-ADC candidates) underscores the emerging status of anti-infective bioconjugates as a novel translational Frontier. Abbreviations: Onc-ADC, oncology antibody-drug conjugate; ID-ADC, infectious disease–targeting antibody-drug conjugate; FDA, U.S. Food and Drug Administration; NTDs, neglected tropical diseases.

**FIGURE 3:**
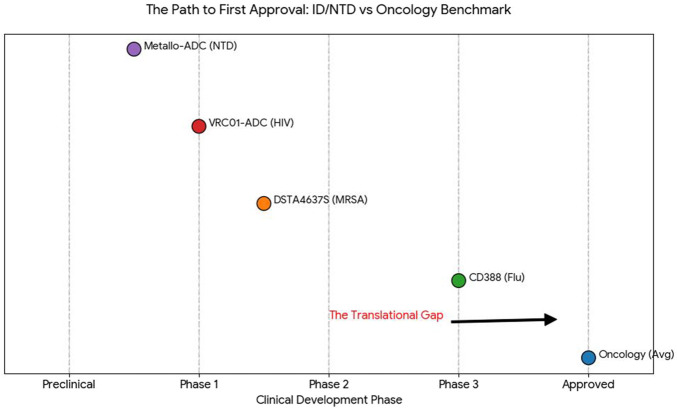
The Path to First Approval: Mapping the Translational Gap between Oncology and Anti-Infective ADCs. This integrative figure quantifies the clinical disparity between the established oncology sector (Onc-ADCs) and the emerging infectious disease-targeting (ID-ADCs) and neglected tropical disease (NTD) landscape over the study period.

The data presented in Table 2 and visualized in Figure 2 (Comparative Analysis of the ADC Global Landscape) and Figure 3 (The Path to First Approval) demonstrate a significant clinical disparity. Despite the “Approved” column for infectious diseases being vacant, the “Phase 3” and “Preclinical” categories are witnessing unprecedented investment in ID-ADCs, indicating a strategic shift in the field from oncology imitation (Onc-ADCs) to pathogen-focused, infection-targeted development.

## 5 Chemistry of linkers for non-oncology ADCs

The linker is an essential element of an antibody-drug conjugate (ADC), serving as the molecular connector that determines the accuracy and effectiveness of therapeutic activity. In oncology, this fuse is intended to be ignited within the global milieu of human lysosomes (Onc-ADCs). Nonetheless, this paradigm is found to be inaccurate and potentially hazardous for infectious diseases. Thus, there is an urgent requirement to develop “smart” linkers that remain inactive throughout systemic circulation but rapidly and selectively cleave upon detecting certain biochemical signals linked to viruses or their infected environments. The transition from a host-centric to a pathogen-centric activation strategy is crucial for attaining the therapeutic window required for successful anti-infective ADCs.

### 5.1 pH-sensitive linkers and infectious microenvironments

Acidic environments are typical of numerous pathogenic processes and offer targetable physicochemical signals for facilitating selective payload release. Conventional acid-labile motifs, such hydrazones and acetals, have been utilized; nevertheless, they frequently exhibit instability in plasma. Next-generation designs seek to enhance the equilibrium between stability and activity. The β-thiopropionate linker exhibits enhanced plasma stability owing to its sterically hindered configuration, while facilitating swift cleavage through intramolecular cyclization in moderately acidic environments (pH 4.5–5.5) commonly present in phagosomes and endosomes (Aoyama et al., 2024). The trimethyl lock lactonization system features a sterically triggered cyclization that is highly sensitive to mild acidification, rendering these linkers suitable for targeting intracellular pathogens such as *Mycobacterium tuberculosis* and Leishmania species, which inhabit acidified phagosomal compartments.

The efficacy of these pH-sensitive linkers is fundamentally reliant on the pronounced pH gradient between the neutral bloodstream and the acidic environments of pathogens, a gradient that is typically more stable and distinct than the fluctuations present in the tumor microenvironment (Onc-ADCs) (Aoyama et al., 2024).

### 5.2 Pathogen-specific enzymatically cleavable linkers

Linkers designed for cleavage by pathogen-specific enzymes exemplify the highest level of selectivity in antibody-drug conjugate design. This strategy directly utilizes the distinctive biochemical characteristics of the pathogen, effectively converting pathogen biology into medicinal chemistry designed for targeted therapeutic applications for ID-ADCs.

#### 5.2.1 GP63-specific linkers (Leishmania)

The zinc-metalloprotease GP63 is highly expressed on the surfaces of Leishmania promastigotes and amastigotes. Linkers that integrate substrate recognition sequences specific to GP63, including oligopeptides with hydrophobic residues, are effectively cleaved upon interaction with the parasite or within its parasitophorous vacuole (Lindesmith et al., 2018). Structural investigations of GP63 have yielded insights that guide the optimization of these sequences for enhanced cleavage kinetics and specificity against host proteases (Lindesmith et al., 2018).

#### 5.2.2 Falcipain-cleavable linkers (malaria)

The cysteine proteases falcipain-2 and -3 from Plasmodium falciparum are essential for hemoglobin degradation and exhibit significant activity in the parasite’s acidic food vacuole. Dipeptide linkers containing amino acids like Arg or Leu in the P2 position (e.g., Leu-Arg) are effectively processed by these enzymes (Zhang Y et al., 2023). This mechanism can be strategically employed in ID-ADCs aimed at infected red blood cells (iRBCs): following internalization and subsequent trafficking, the linker is cleaved by falcipains, thereby releasing the therapeutic payload within the parasite (Zhang, Y et al., 2023).

#### 5.2.3 HIV protease-cleavable linkers

The HIV-1 protease is a crucial viral enzyme necessary for replication, present in elevated concentrations within the cytoplasm of productively infected cells. Linkers that encode the protease’s native cleavage sites, obtained from the Gag-Pol polyprotein, can be employed to construct ID-ADCs that specifically deliver their payload to infected cells. This method incorporates a biological rationale, guaranteeing that payload activation predominantly transpires in cells that are actively facilitating viral replication (Majumder et al., 2024; Savoy et al., 2021).

### 5.3 Reactive oxygen species-responsive linkers in inflamed tissues

Chronic infections and inflammatory conditions are generally marked by oxidative stress, leading to increased levels of reactive oxygen species (ROS), such as hydrogen peroxide (H_2_O_2_). This dynamic offers a unique chemical trigger independent of enzymatic activity. Arylboronic esters serve as prominent structures for ROS-sensitive release; upon oxidation by H_2_O_2_, the arylboronic acid transforms into a phenol, which swiftly undergoes 1,6-elimination to liberate the payload. Thioketal linkers are likewise cleaved by reactive oxygen species (ROS), yielding a ketone and thiols (Chuprakov et al., 2021). These mechanisms are especially relevant to diseases characterized by pronounced granulomatous inflammation, such as tuberculosis and specific fungal infections, wherein an ID-ADCs can maintain stable circulation while swiftly releasing its payload upon entering the reactive oxygen species (ROS)-rich core of the lesion, thereby ensuring accurate spatial control (Johannes et al., 2021; Schmitt et al., 2023).

### 5.4 Structural insights into linker stability and cleavage mechanisms

The design of rational linkers is closely associated with structural biology and computational chemistry. Molecular docking and dynamics simulations are crucial methodologies for elucidating the interactions between a candidate peptide linker and the active sites of pathogen proteases such as GP63 or falcipain. These simulations facilitate the prediction of binding modes, the calculation of interaction energies, and the guidance of modifications to improve cleavage rates and specificity. Computational studies can model oxidation and elimination pathways for ROS-sensitive linkers, offering insights for the design of more stable or rapidly responsive variants. Moreover, simulating the entire ADC structure can reveal whether the linker is solvent-exposed or embedded inside the antibody surface, so directly affecting its stability in plasma and accessibility to the activating trigger. This integrated structural and computational methodology converts linker design for ID-ADCs from empirical trial-and-error process into a predictive engineering discipline, essential for the advancement of the next-generation of infection-responsive ADCs (Chuprakov et al., 2021; Marei et al., 2022; Mondal et al., 2018; Zhang, D et al., 2018).

The diverse chemical strategies for achieving selective payload release in infectious disease settings are summarized in Table 3, which catalogs linker classes, their activation triggers, and representative applications across ID/NTDs.

**TABLE 3:** Antibody-drug conjugate (ADC) linker technologies: triggers and applications (2018–2025).

Linker class	Specific example	Application (ID/NTD)	Trigger mechanism	Trigger conditions (pH/Enzyme)	References
Acid-Labile	Hydrazone	*S. aureus* (MRSA), *T. brucei*	Hydrolysis in acidic compartments	pH 4.5–5.5 (Lysosome/Endosome)	Baah et al. (2021), Iwamoto et al. (2023)
Acid-Labile	Orthoester (HMPO)	Universal (Pro-drug platform)	Acid-mediated 1,6-elimination	pH 5.5 (Infection/Inflammation site)	
Protease-Cleavable	Val-Cit (Dipeptide)	*S. aureus* (DSTA4637S)	Cleavage by host or bacterial proteases	Cathepsin B (Lysosomal)	Cai et al. (2020), Peck et al. (2019)
Protease-Cleavable	Gly-Gly-Phe-Gly	Viral/Bacterial (High-load)	Sequential peptide cleavage	Lysosomal Proteases	
Pathogen-Specific	Penicillin-G	Gram-negative bacteria	Enzymatic hydrolysis by $\beta$-lactamases	beta-lactamase (Bacterial specific)	Homer et al. (2024)
Pathogen-Specific	Sugar-based	Leishmaniasis/Malaria	Cleavage by parasite glycosidases	beta-Glucuronidase	
Redox-Responsive	Disulfide	Intracellular Parasites	Thiol-disulfide exchange (Glutathione)	High GSH (Cytoplasmic/Intracellular)	Liu et al. (2020), Motley et al. (2019)
Non-Cleavable	Thioether (MCC)	HIV/Chronic Infections	Total antibody degradation	Proteolysis (Complete cellular digestion)	Döhrmann et al. (2024)

## 6 ADC payloads for infectious diseases: advancing beyond cytotoxicity

The therapeutic aim of an infectious disease ADC (ID-ADC) requires a fundamental rethinking of the payload’s function. In contrast to oncology ADCs (Onc-ADCs), which seeks to eliminate target human cells, the objective here is the selective eradication of the pathogen while ideally preserving the host cell. This paradigm shift necessitates a transition from the highly potent cytotoxins that prevail in cancer therapy to a varied array of payloads with mechanisms specifically designed for microbial physiology. The augmentation of this arsenal signifies a pivotal and new Frontier in the domain, including targeted antimicrobials, immunomodulators, host-directed medicines, and novel chemotypes.

This paradigm shift necessitates a transition from cytotoxins to a diverse array of non-cytotoxic payloads with mechanisms specifically tailored to microbial physiology, as summarized in Table 2.

### 6.1 Antimicrobial payloads

A fundamental approach in the development of infectious disease antibody-drug conjugates (ADCs) is the employment of current antibiotics or their derivatives as payloads. This method aims to enhance the pharmacokinetics and distribution of established medications by conjugating them to antibodies designed for certain illnesses. For example, associating an antibiotic such a rifamycin derivative with targeting *Staphylococcus aureus* illustrates how these conjugates can attain localized high concentrations, overcoming the difficulties presented by biofilms and inadequate uptake into infected host cells (Yu et al., 2022). Incorporating traditional antibiotics into ID-ADC designs improves targeted delivery and seeks to mitigate the systemic toxicity frequently linked to broad-spectrum antibiotics (Matsuda et al., 2025).

The difficulty involves altering intricate antibiotic structures to integrate stable linkers while preserving their pharmacophoric characteristics. The evolution of linker chemistry that preserves drug integrity while facilitating attachment is essential, as evidenced by progress in linker design (Neumann et al., 2018). Understanding drug-target interactions guides the modifying process to maintain the drug’s efficacy (Balamkundu and Liu, 2023b).

### 6.2 Immune-modulating payloads

ADCs can utilize immune-modulating payloads that augment the host’s immune response to infections rather than directly eradicating the germs. For instance, agonists of Toll-like receptors (TLRs) or STING pathway activators can be precisely administered to infected cells. This targeted administration enhances localized immune responses, converting an immunosuppressive environment into one that actively combats the pathogen while reducing the likelihood of systemic side effects (Mukherjee et al., 2018). This targeted immune regulation depends significantly on the design of payloads that elicit a strong response, highlighting the equilibrium between efficacy and safety in their use (Yelamali et al., 2024).

### 6.3 Host-directed therapeutic payloads

Host-directed therapy (HDT) is an innovative strategy that focuses on host characteristics critical for the survival and reproduction of pathogens. This technique reduces the likelihood of resistance emergence by utilizing non-mutating host cell mechanisms. Efficient HDT payloads comprise autophagy modulators and small compounds that interfere with pathogen-adaptive mechanisms by targeting host kinases (Ngambenjawong et al., 2022). The precision of ADCs in targeting these therapeutics to the infectious microenvironment guarantees enhanced treatment effectiveness with diminished off-target effects, requiring thorough understanding of host-pathogen interactions (O’Leary et al., 2023).

### 6.4 Attachment chemistries: preserving payload integrity and potency

The design of the linker-payload interface is a crucial factor in therapeutic efficacy. Neumann et al. (2018) emphasize that a linker must preserve systemic stability while ensuring that its attachment does not undermine the structural integrity or biological efficacy of the medication. This has been accomplished in the current landscape using three principal chemical strategies: bio-orthogonal “click” handles, self-immolative spacers, and structural silencing of non-essential functional groups.

The strategic approaches to conjugate these diverse payloads while preserving their integrity are compared in Table 4.

**TABLE 4:** Attachment strategies for drug integrity in antibody-drug conjugates.

Strategy	Attachment mechanism	Impact on drug integrity	Primary application in ID/NTDs	References
Bio-orthogonal click	Azide-Alkyne (SPAAC)	High: Precision attachment at non-interfering sites	Developing effective ADCs for targeting resistant viral strains	Matveev et al. (2019)
Self-immolative	PAB/Val-Cit Spacers	High: Drug is released in its native, unmodified state	Payloads sensitive to steric hindrance	Wang et al. (2024)
Thiol-maleimide	Cysteine-specific	Moderate: Relies on available sulfur groups; highly stable	Traditional antibacterial payloads and certain ADCs	Yuan et al. (2019)
Enzymatic tagging	Glycan/MTGase	High: Uses antibody carbohydrates to avoid protein interference	Large-molecule proteins or catalytic metallo-complexes	Pericolini (2018)

#### 6.4.1 Bio-orthogonal and site-specific attachment

Conventional stochastic conjugation to lysine or cysteine residues often resulted in heterogeneous drug-to-antibody ratios and potential masking of the payload’s active site. Modern ID-ADC methodologies employ bio-orthogonal chemistry, such as Strain-Promoted Azide-Alkyne Cycloaddition (SPAAC). By introducing an azide “handle” at a non-pharmacophoric position on the payload, the linker can be precisely attached via a “click” reaction. This approach preserves the native structure and pharmacophore of the therapeutic agent because the bio-orthogonal functional groups are inert and absent from both human and pathogen proteomes, ensuring minimal interference with drug activity. This conjugation strategy is illustrated in Equation 1.

$$\text{Payload}–N_{3}+\text{Cyclooctyne}–\text{Linker }\to \text{ Payload}–\left(1,2,3-\text{triazole}\right)–\text{Linker}$$ (1)

#### 6.4.2 The role of self-immolative spacers

For payloads that diminish efficacy with modest structural alterations, self-immolative spacers (e.g., p-aminobenzyloxycarbonyl or PAB) function as a chemical “disappearing act.” These spacers are positioned between the cleavage site and the drug. When activated by a pathogen-specific enzyme or a pH change, the spacer undergoes a spontaneous 1,6-elimination or cyclization cascade. This leads to the liberation of the original, unaltered medication into the pathogen’s microenvironment, guaranteeing that the warhead attains its target with complete restoration of its native integrity. The general structure of such linker–spacer–payload systems is represented in Equation 2.

$$\text{Linker}–\text{NH}–\text{CO}–O–{\text{CH}}_{2}–\left(p-\text{phenyl}\right)–\text{NH}–\text{CO}–\text{Payload}$$ (2)

#### 6.4.3 Structural silencing and interaction mapping

Advancements in drug-target interaction mapping, facilitated by AI-driven docking and high-resolution cryo-electron microscopy, enable the intentional “silencing” of binding sites. By pinpointing the precise “face” of the medication that engages with the pathogen’s protein (the pharmacophore), chemists can choose an attachment site on the corresponding, “silent” side. This guarantees that the substantial antibody-linker complex does not sterically impede the drug’s capacity to bind its target upon internalization. The general structure of such linker–spacer–payload systems is represented in Equation 3.

$$\text{Solvent}/\text{Linker exposed}\leftarrow \;\left[\text{Silent face}\right]—\left[\text{Active face}\right]\to \text{ binds protein}$$ (3)

$$\text{Payload}–\text{NH}–\text{CO}–\text{Linker};\text{ Payload}-O-\text{CO}-\text{Linker};\text{ Payload}–{\text{CH}}_{2}–O–\text{Connector}$$

### 6.5 Structural biology and payload-target interactions

The successful integration of novel payloads into ID-ADCs fundamentally relies on structural biology, which enables rational design by elucidating specific interactions between the payload and its target. High-resolution structural studies of antibiotic-target complexes can inform the strategic positioning of linkers while maintaining binding efficacy (Fu et al., 2022). This rational design strategy transitions ID-ADC creation from conventional trial-and-error to a systematically engineered methodology, crucial for creating effective therapeutic medicines against infections (Tonon et al., 2024).

It is essential to transition to new chemotypes that investigate bioinorganic compounds as prospective next-generation ID-ADC payloads to combat adaptable infections. Metallo-drug payloads present distinct modes of action; yet, their development encounters considerable hurdles concerning stability and conjugation optimization (Tvilum et al., 2023). Ongoing research in ADC technology is yielding potential advancements in innovative therapeutic options for combating infectious infections.

## 7 Metallo-drugs as emerging ADC payloads

The necessity to create innovative anti-infective agents that evade known resistance pathways requires investigation beyond conventional organic chemistry (Wang et al., 2023). Bioinorganic complexes, metallo-drugs, present a fundamentally unique and predominantly unexplored chemical domain. Distinguished by distinctive three-dimensional structures, complex redox chemistry, and modes of action that diverge from traditional antibiotics, metallo-drugs offer an intriguing avenue for advancements in ID-ADC payloads (García-Alonso et al., 2018; Yamazaki et al., 2021). Their incorporation into specialized delivery systems signifies a collaborative fusion of synthetic inorganic chemistry, medicinal design, and pathogen biology, with the capacity to provide a novel category of precision treatments for resistant diseases.

**FIGURE 4:**
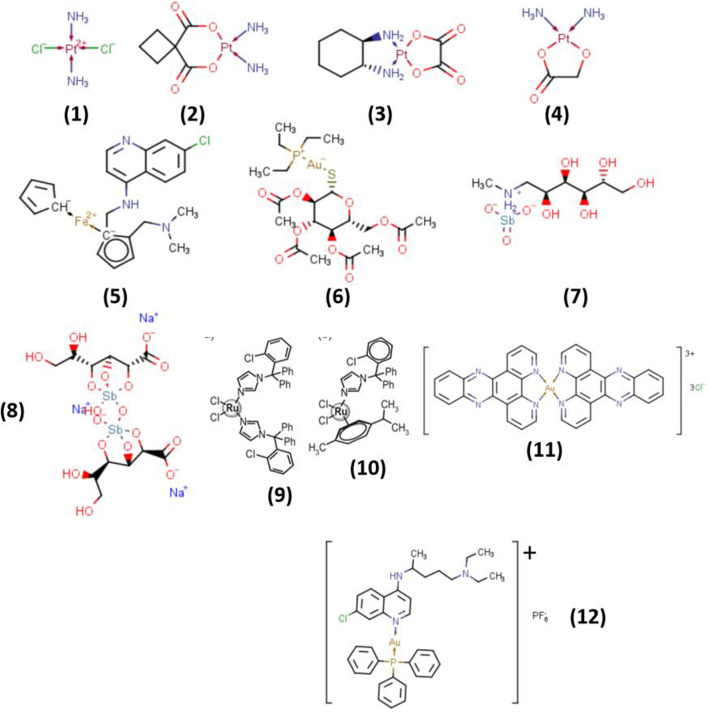
Selected metallo-drug Payload Architecture that are currently used clinically for cancer (1, 2, 3, 4), and leishmaniasis (7, 8), and in the experimental clinical phase for malaria (5), Selected metal-based therapeutic agents for trypanosomiasis (9, 10), methicillin-resistant *Staphylococcus* (6) leishmaniasis (11) and malaria (12).

This improvements have enabled the development of a wide range of metal-containing therapies, several of which have moved to clinical usage, demonstrating the translational feasibility of metallo-drugs as prospective ID-ADC payloads. As demonstrated in Figure 4, platinum-based complexes—including cisplatin (1), carboplatin (2), nedaplatin (3), and oxaliplatin (4), remain fundamental to ONC-ADC chemotherapy. Beyond oncology, gold complexes such as auranofin (6) have proven antibacterial efficacy, while antimony-based medicines, notably meglumine antimoniate (7) and sodium stibogluconate (8), are established therapies for leishmaniasis. In parallel, metal-based agents derived from ruthenium, iron, and platinum continue to advance through experimental clinical evaluation for cancer, and ferroquine (5) has reached clinical testing for malaria (Figure 4) (Nriagu and Skaar, 2015).

Metal coordination can markedly augment the biological activity of established chemical ligands. Clotrimazole (CTZ) and ketoconazole (KTZ) display intrinsic antiparasitic action against Trypanosoma cruzi; however, their efficiency is considerably increased upon complexation with metals such as ruthenium, copper, rhodium, platinum, and gold. Notably, RuCl_2_(CTZ)_2_ (9) and Ru(η^6^-p-cymene)Cl_2_(CTZ) (10) display much stronger inhibition of T. cruzi epimastigote growth than the free CTZ and KTZ ligands. Similarly, the gold complex [Au(dppz)_2_]Cl_3_ (12) demonstrates improved leishmanicidal activity (Nriagu and Skaar, 2015).

These examples highlight how strategic chemical modifications of payloads, including redox-active metallo-drugs, directly satisfy the unique ID-ADC design criteria, such as selective pathogen lethality, stability in systemic circulation, and compatibility with pathogen-responsive linkers, distinguishing them from conventional oncology ADC payloads. These examples highlight how strategic chemical modifications of payloads, including redox-active metallo-drugs, directly satisfy the unique ID-ADC design criteria, such as selective pathogen lethality, stability in systemic circulation, and compatibility with pathogen-responsive linkers, distinguishing them from conventional oncology ADC payloads. Collectively, these instances demonstrate the molecular diversity, clinical precedence, and potency amplification achievable through metal coordination. When translated into ID-ADC architectures, metallo-drugs combine target specificity with metal-driven modes of action, presenting them as attractive payload options for next-generation anti-infective and ONC-ADC therapies. The mechanistic diversity and clinical precedent of metallo-drugs are presented in Figure 4, which exhibits selected architectures from clinically used medications to experimental candidates, demonstrating their potential as modular ID-ADC payloads.

### 7.1 Rationale for using metal complexes in ADCs

The impetus behind integrating metallo-drugs into ID-ADC frameworks originates from their various mechanistic paths, which are often unreachable by organic molecules. For instance, metallo-complexes such as those containing iron or copper can catalyze processes that form reactive oxygen species (ROS) through Fenton-type chemistry. This potential can be leveraged to destroy pathogen cells directly within their niche, employing the altered redox conditions typical of infections (Pan et al., 2019). Furthermore, these metal complexes can disrupt genetic material by intercalating into or altering pathogen DNA/RNA structures, causing to replication failures or fatal mutations (Sun et al., 2025).

Additionally, the unusual physicochemical features of metallo-drugs allow them to evade existing pathogen resistance pathways. For instance, infections generally possess efflux pumps that eject organic medications; yet, the variable charge and size of metal complexes can bypass these defenses (Meng et al., 2024). Furthermore, the tunability of metal coordination permits rational design targeted to target certain biological vulnerabilities by altering redox potentials, lipophilicity, and stability (D’Agostino et al., 2020).

### 7.2 Structural features of tetraaza macrocycles and phenanthroline systems

Among the classes of metallo-drug payloads, tetraaza macrocycles and phenanthroline-based systems stand out for their prospective applications inside ID-ADCs. Tetraaza complexes, such as those based on cyclam, give significant kinetic inertness in biological settings. This characteristic guarantees that the hazardous metal remains bound until it reaches its intended target, minimizing off-target effects that can come from premature release (Jiang Y et al., 2024). The capacity of these complexes to create stable contacts with polyanionic biological materials, such as DNA or microbial surfaces, highlights their potential as targeted therapeutic agents.

On the other hand, phenanthroline-based complexes offer a significant advantage due to their aptitude for DNA intercalation. The metal ion (e.g., Cu or Fe) can be selected based on the desired action—redox-active metals can increase oxidative DNA cleavage, whereas transition metals like nickel can hinder critical enzyme processes (Giese et al., 2021). Such tactics boost the therapeutic efficacy of the ID-ADCs by exploiting numerous mechanisms against pathogenic pathogens, therefore complicating the development of resistance (Yang et al., 2021).

### 7.3 Mechanistic basis: ROS production, DNA binding, and enzyme inhibition

The therapeutic benefits of metallo-drug payloads in ID-ADCs stem from three basic mechanisms: ROS production, DNA binding/cleavage, and enzyme inhibition. The creation of ROS is particularly effective against pathogens residing within phagocytic cells, where the redox environment can be exploited to induce localized oxidative stress (Xu et al., 2022). This is paired with the ability for metallo-drugs to bind to and break pathogen DNA, leading in interruption of critical processes such as replication, especially in fast proliferating bacterial or parasite infections (Yamazaki et al., 2024).

Moreover, metallo-drugs can also serve as transition-state analogs or act as inhibitors that displace critical metals from enzymes required for pathogen survival. Such tactics ensure a multi-target approach to disruption, significantly lowering the probability of resistance evolution (Lee et al., 2022). By harnessing the unique qualities of metallo-drugs, researchers can boost the effectiveness and specificity of ID-ADCs in battling illnesses that are increasingly challenging to treat with traditional medicines.

### 7.4 Potential application in TB, leishmaniasis, HIV, and malaria

The distinctive qualities of metallo-drugs correlate closely with the therapeutic needs of various high-burden infectious illnesses such as tuberculosis (TB), leishmaniasis, HIV, and malaria. These diseases present significant pathophysiological obstacles, making them great candidates for new metallo-drug methods within the ID-ADC framework.

Tuberculosis (TB) offers a tough challenge due to the peculiar hypoxia and oxidative stress-prone environment of tuberculous granulomas. Redox-active chemicals have been explored for their ability to selectively target niches within *Mycobacterium tuberculosis*, triggering deadly DNA damage (Basarab et al., 2022). The selective action of these metallo-drugs holds promise for boosting the efficacy of existing TB medicines, allowing for focused intervention that mitigates off-target symptoms often associated with traditional therapies (Basarab et al., 2022).

In the context of leishmaniasis, metallo-drugs can exploit specific enzymatic weaknesses such as the zinc protease GP63, which is crucial for the survival and reproduction of Leishmania parasites. Designing metallo-drug payloads that inhibit this enzyme or harness it for controlled release through linker cleavage may provide potent selective targeting and treatment of leishmaniasis (Krokhotin et al., 2019). This dual method optimizes payload delivery efficacy while lowering the chance of developing resistance.

Metallo-drugs offer a novel approach to target the integrated proviral DNA within latent reservoirs of HIV, where traditional antiretroviral medications frequently prove ineffective (Pincus et al., 2025). Metallo-drugs that can impair latent DNA or destroy reservoir cells offer a novel approach for HIV eradication techniques, potentially overcoming a significant obstacle in HIV treatment (Pincus et al., 2025). This mechanistic approach highlights the growing emphasis on innovative medicines capable of penetrating deeper into infection reservoirs.

Metallo-drugs show potential in tackling the issues presented by artemisinin-resistant pathogens in the fight against malaria. Complexes engineered to induce oxidative damage in the feeding vacuole of Plasmodium or impede heme detoxification can interfere with essential survival mechanisms of the malaria parasite, signifying a novel approach in malaria treatment (Su et al., 2018). By focusing on distinct metabolic pathways exclusive to the parasite, these metallo-drugs can markedly diminish the probability of resistance emergence in therapeutic protocols (Murugesan et al., 2018).

The translational potential of metal-based compounds for key infectious diseases is cataloged in Table 5, while Table 6 provides a direct comparative analysis of metallo-drugs against their organic counterparts, underscoring advantages in potency and resistance evasion.

**TABLE 5:** Overview of metal-based compounds for infectious diseases.

Compound/Class	Target disease	Mechanism(s)	Status	References
Ferroquine (FQ, SSR97193)	Malaria (Plasmodium falciparum)	Organometallic ferrocene-chloroquine derivative; likely inhibits heme detoxification/hemozoin formation; active against chloroquine-resistant strains	Phase II clinical trials completed/ongoing for uncomplicated P. falciparum malaria	Matveev et al. (2019), Wang et al. (2024)
Auranofin (Gold-based, Au(I))	HIV reservoir reduction, TB host-directed therapy, Leishmania	Inhibits thioredoxin reductase (TrxR) and disrupts redox homeostasis; proposed effects on viral reservoirs and parasitic redox systems	Approved for rheumatoid arthritis; repurposing in clinical trials for HIV reservoir reduction; Phase II TB trial (status unclear)	Yuan et al. (2019)
Sodium stibogluconate	Leishmaniasis	Metal (Sb)-containing therapy that interferes with parasite metabolism and enzyme systems	Approved/widely used as a mainstay treatment for cutaneous and visceral leishmaniasis (resistance/toxicity issues persist)	Pericolini (2018)
Antimony-based drugs (other salts)	Leishmaniasis	Similar action to sodium stibogluconate; antiprotozoal metal action	Approved/clinical use (pentavalent antimonials)	Amaral et al. (2019)
Gold complexes (e.g., Au–chloroquine complexes)	Malaria (*in vitro*), leishmaniasis (*in vitro*)	Metal coordination to chloroquine scaffold; suggests improved binding to parasite heme and DNA interaction	Preclinical—*in vitro*/early animal studies	Fujita et al. (2019)
Ruthenium/Rhodium-chloroquine complexes	Malaria (chloroquine-resistant strains)	Metal improves efficacy and overcomes resistance; potential prohibition of hemozoin formation	Preclinical (*in vitro* and animal)	Tateishi et al. (2025)
Gold nanoparticles/Au complexes	Leishmania (preclinical)	Nanoparticle forms may interact with parasite redox and DNA; potential antiparasitic effects observed *in vitro*	Preclinical	Gautam et al. (2024)
Copper, ruthenium, platinum, zinc complexes (various)	Trypanosomatid parasites, Leishmania, Malaria	Coordinate to ligands that disrupt parasite DNA or metabolic pathways	Preclinical research	

**TABLE 6:** Comparative overview of metal-based and organic compounds for treating infectious diseases.

Disease	Compound/Drug	Type	Activity	Mechanism summary	References
Malaria (Plasmodium falciparum)	Ferroquine (SSR97193)	Metallo-drug (ferrocene-quinoline)	IC_50_ ∼ 9.3 nM (multi-drug resistant isolates); ∼36× more potent than chloroquine in one study	Inhibits hemozoin formation; accumulates in the digestive vacuole; active against chloroquine-resistant strains	Matveev et al. (2019) , Wang et al. (2024)
​	Chloroquine (CQ)	Standard organic antimalarial	IC_50_ ∼ 340.8 nM (resistant isolates)	Blocks β-hematin formation; released by PfCRT-mediated efflux in resistant parasites	Yuan et al. (2019)
​	Ferroquine vs. CQ resistant isolates	Comparison	Ferroquine ∼36-fold more potent vs. CQ	Ferroquine is active even against resistant strains	Pericolini (2018)
​	—	Ferroquine metabolite (SR97213A)	IC_50_ ∼ 37 nM (less active than parent)	Metabolite retains antiplasmodial activity	Amaral et al. (2019)
Leishmaniasis (Leishmania major)	Auranofin	Gold(I) repurposed metallo-drug	IC_50_ ∼ 1.007 μg/mL (∼2.8 µM) against amastigotes; promastigotes ∼2.38 μg/mL (∼6.7 µM)	Inhibits trypanothione reductase causing oxidative stress and parasite death	Fujita et al. (2019)
​	Sodium stibogluconate	Approved antimonial drug	Standard comparator with broad use; exact IC_50_ varies but widely effective clinically	Interferes with parasite metabolism; reduces ATP/GTP synthesis	Tateishi et al. (2025)
​	Miltefosine	Standard organic anti-leishmanial	IC_50_ generally lower than antimonials in some studies (<5 µM typical)	Disrupts parasite lipids and signaling	Gautam et al. (2024)
​	Gold complexes (preclinical)	Gold(I) complexes	IC_50_ 0.5–5.5 µM against intracellular amastigotes (various species)	Inhibits trypanothione reductase; causes ROS and mitochondrial damage	​
HIV/TB	Auranofin (repurposed)	Metallo-drug	Investigational activity in HIV reservoir reduction and TB host-targeted research	Inhibits redox enzymes; induces oxidative stress	Yuan et al. (2019)

### 7.5 Conjugation challenges and opportunities

The incorporation of metallo-drugs into ID-ADC frameworks presents synthetic problems that require inventive solutions. A significant problem is attaining stable conjugation while maintaining the intrinsic characteristics of the metal complex. Conventional bioconjugation techniques utilizing thiols or amines may result in competition with endogenous donors for binding to the metal center, potentially causing trans-chelation and compromising the integrity of the metal ion during conjugation (Zong et al., 2020).

To address these challenges, it is essential to develop ligand scaffolds incorporating bioorthogonal conjugation sites, such as azides or alkynes appropriate for copper-free click chemistry, that are positioned away from the coordination sphere. This separation method can improve the stability of the metallo-drug, guaranteeing good distribution upon activation (Walter et al., 2022). Alternatively, formulating the metallo-drug as a prodrug, wherein the linker functions as an auxiliary ligand, may enable pathogen-specific cleavage that activates the drug solely within the disease milieu. This method guarantees regulated release and may facilitate alterations in the drug’s coordination geometry or redox state, thus transforming an inert chemical into an active form (Usuda et al., 2021).

Effectively addressing these conjugation problems will allow researchers to harness the complete potential of metallo-drugs as novel payloads in ID-ADC techniques, thus expanding the treatment alternatives for persistent infectious illnesses.

### 7.6 Coordination chemistry and mechanistic versatility of metallo-pharmaceutical payloads

The shift from organic cytotoxins to metallo-drug payloads introduces a novel aspect of therapeutic regulation: geometric and electrical tunability. In contrast to organic molecules, the reactivity of a metal complex can be accurately adjusted by altering the metal center, its oxidation state, and the configuration of surrounding ligands.

#### 7.6.1 Structural scaffolds: macrocycles and polypyridyl complexes

The advancement of ID-ADC has progressively concentrated on refining the structural scaffolds employed for payloads. Two notable scaffolds have garnered attention for their significant thermodynamic stability and kinetic inertness in systemic circulation:

##### 7.6.1.1 Tetraaza macrocycles (e.g., cyclams)

Tetraaza macrocycles, including cyclams, function as essential ligands that stabilize transition metals such as Ni^2+^ or Cu^2+^. These immobilized metal complexes are frequently engineered with “pendant arms” that enhance bioconjugation with antibodies. The distinctive mechanism of action for these complexes is the potential to produce localized reactive oxygen species (ROS) via processes similar to Fenton chemistry in pertinent settings. The resilience of cyclams is notable, as they may endure systemic circulation, so guaranteeing the intact delivery of the payload to the designated target (Aoyama et al., 2024; Petersen et al., 2024; Shia et al., 2025).

##### 7.6.1.2 Polypyridyl complexes (e.g., Ru(bpy)_3_^2+^ or Cu(phen)_2_^2+^)

Polypyridyl complexes, distinguished by their octahedral or square-planar geometry, are acknowledged for their potential DNA intercalation capabilities. When coupled to antibodies, these complexes may provide tailored delivery techniques. Nonetheless, evidence about their direct effectiveness against multi-drug resistant (MDR) bacteria remains a field of active investigation, and assertions regarding irreversible DNA strand cleavage require validation through additional investigations (Cazzaniga et al., 2025; Chuprakov et al., 2021; Shia et al., 2025). The emphasis on structural scaffolds that augment stability is essential for enhancing treatment efficacy against drug-resistant bacteria.

#### 7.6.2 The “linker-metal” interface

A significant problem in the creation of ID-ADCs pertains to the attachment site of the linker to the metal complex. To guarantee the stability of the metal complex during circulation, it is essential that the linker binds to the ligand backbone instead of the metal core. This configuration markedly diminishes the probability of premature metal dissociation prior to arriving at the designated target (Petersen et al., 2024; Savoy et al., 2021; Wang et al., 2019). Innovative methodologies, including click-chemistry approaches such as Strain-Promoted Azide-Alkyne Cycloaddition (SPAAC), facilitate the accurate conjugation of azide-functionalized macrocycles to alkyne-tagged antibodies, maintaining the stability of the metal complex until it reaches its intended destination (Savoy et al., 2021; Schmitt et al., 2023).

The primary benefits of metallo-drugs compared to conventional antibiotics, together with instances of innovative complexes, are encapsulated in Tables 7, 8, underscoring their justification as a next-generation class of therapeutics for ID-ADCs.

**TABLE 7:** Comparative advantages of metallo-drug over organic antibiotics in infectious disease treatment.

Feature	Organic antibiotics (e.g., Penicillin, Rifampicin)	Metallo-Drugs (e.g., Ru-Complexes, Cu-Cyclams)	References
Mechanism	Stoichiometric: Binds 1:1 to a specific protein (Enzyme/Ribosome)	Multimodal: Can act through multiple pathways, including the generation of ROS.	Jaafar et al. (2020), Llamazares et al. (2019)
Geometry	Primarily Planar/2D; limited by carbon-carbon bond angles	Octahedral/3D: Unique 3D shapes may enhance penetration through bacterial membranes	Hilst et al. (2019), Shekhar et al. (2022)
Resistance	High risk: Single-point mutations can confer resistance	Lower risk: Their ability to target multiple sites reduces the chance of resistance emergence	Omar et al. (2025), Zabara et al. (2021)
Redox Activity	Largely redox-inactive; standard antibiotics do not primarily rely on redox reactions	Often redox-active: Some metallo-drugs can be activated under specific conditions to increase their antibacterial activity	Abdel-Rahman et al. (2024), Sovari and Zobi (2020)
Selectivity	High (Target-specific)	Variable (Careful ligand design is essential to minimize host toxicity)	Biji et al. (2021), Huang et al. (2023)

**TABLE 8:** Pioneering metallo-drugs in infectious diseases and neglected tropical diseases.

Metal	Example complex	Pathogen/Disease	Mechanism of action
Antimony (Sb)	Sodium Stibogluconate	Leishmaniasis (NTD)	Inhibits trypanothione reductase; disrupts parasite bioenergetics
Arsenic (As)	Melarsoprol	African Trypanosomiasis	Irreversibly binds to pyruvate kinase; blocks parasite glycolysis
Silver (Ag)	Silver Sulfadiazine	Wound/Burn Infections	Multiple: DNA binding, cell wall disruption, and protein denaturation
Bismuth (Bi)	Pravibismane (MBN-101)	Biofilm Infections/DFI	Disrupts bacterial cell surface and inhibits biofilm matrix production
Ruthenium (Ru)	Ferroquine-Ru Hybrids	Malaria/MDR-TB	Intercalates DNA; blocks heme detoxification in parasites
Copper (Cu)	Cu-Cyclam Complexes	Leishmaniasis/MRSA	Catalytic: Fenton-like ROS generation leads to oxidative damage
Gold (Au)	Auranofin	Amoebiasis/TB	Inhibits thioredoxin reductase; induces lethal oxidative stress

## 8 Computational design pipeline for infectious disease ADCs

The rational development of infectious disease ADCs increasingly relies on computational pipelines that replace empirical screening with predictive, multi-scale design. Structural bioinformatics, molecular simulations, and AI-driven modeling collectively enable systematic optimization of antigens, antibodies, linkers, and payloads, accelerating translational readiness while reducing experimental burden. These computational and structural approaches exemplify a design-driven paradigm, where antigen selection, epitope accessibility, and linker positioning are optimized specifically for infectious microenvironments, rather than relying on empirical oncology-focused strategies.

### 8.1 Structural bioinformatics for antigen discovery

Computational surfaceome analysis enables the prioritization of ID-ADC-suitable pathogen antigens by identifying proteins bearing signal peptides, transmembrane domains, or GPI anchors, followed by conservation analysis across clinical strains (Baalmann et al., 2019; Choi P. J. et al., 2020). Structural prediction of surface-exposed loops further refines epitope selection by identifying geometries favorable for antibody engagement, reducing large proteomes to experimentally tractable target sets (John et al., 2018; Trail et al., 2023).

### 8.2 AI-driven antibody and antigen complex design

Advances in protein structure prediction, including AlphaFold2, enable accurate modeling of pathogen antigens lacking experimental structures, facilitating epitope mapping for highly variable targets such as malaria and HIV (Gui et al., 2019; Suzuki et al., 2023). Generative tools such as RFdiffusion allow *de novo* antibody or nanobody design against defined epitopes, while *in silico* affinity maturation optimizes binding strength within ranges compatible with ID-ADC internalization and trafficking (Market et al., 2022; Shim, 2020).

### 8.3 Molecular dynamics for linker stability and cleavage

All-atom molecular dynamics simulations assess the conformational stability and solvent exposure of IgG–linker–payload assemblies under physiological conditions, identifying linkers prone to premature cleavage (Singh et al., 2019). Docking and MD studies further enable the rational design of pathogen-responsive linkers for ID-ADCs by modeling interactions with pathogen proteases such as Leishmania GP63 while minimizing off-target cleavage by host enzymes (Cazzaniga et al., 2025; Cheng et al., 2019).

### 8.4 QSAR/QSPR and docking for payload optimization

QSAR and QSPR modeling correlate physicochemical and quantum-derived descriptors of antimicrobial and metallo-drug payloads with biological activity and cytotoxicity, guiding iterative payload refinement for ID-ADC (Lu et al., 2022). Molecular docking identifies linker attachment sites that preserve target engagement, while elucidating DNA or enzyme binding modes relevant to both organic antibiotics and metal-based payloads (Maciel et al., 2024).

### 8.5 Systems-level and multi-scale modeling

**FIGURE 5:**
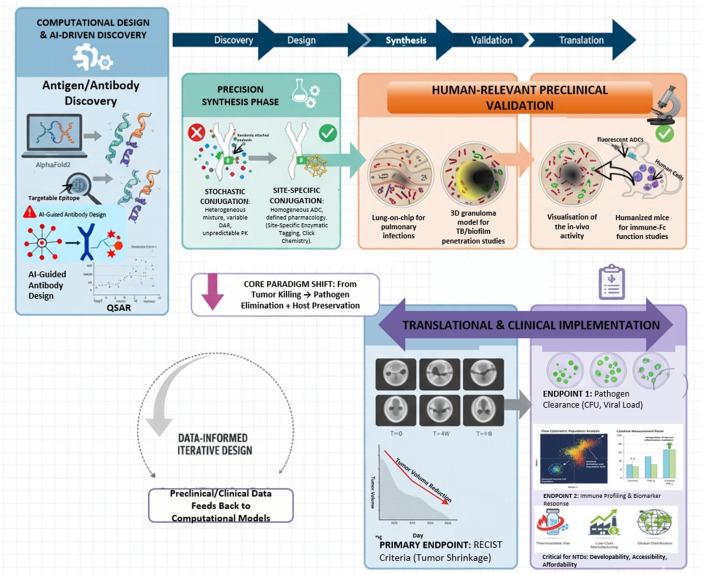
The Integrated Translational Pipeline for Infectious Disease ADC Development. This multidisciplinary roadmap illustrates the end-to-end framework required to transition Antibody-Drug Conjugates (ADCs) from AI-driven discovery to clinical implementation, highlighting the systemic shift from oncology paradigms to pathogen-specific strategies.

Multi-scale PK/PD models integrate ID-ADC binding, internalization, linker cleavage, payload release, and pathogen response into predictive frameworks that simulate *in vivo* efficacy (Erhardt et al., 2018). Sensitivity analyses identify dominant design parameters governing therapeutic outcome, while microenvironmental modeling captures the impact of pH, oxygen tension, and nutrient gradients within granulomas or biofilms (Fidler et al., 2019; Umezaki et al., 2022). Together, these approaches unify chemical design and biological response into a coherent translational pipeline (Figure 5).

## 9 Advanced models for ADC evaluation: integrating chemical design and translational efficacy

The effective development and validation of antibody-drug conjugates (ID-ADCs) aimed against infectious diseases depend on proving their selective efficacy in settings that effectively represent human pathophysiology. Conventional models, including two-dimensional monocultures and routine animal testing, frequently do not accurately mimic the intricate spatial structures and dynamic metabolic gradients present in real infection sites. To address these constraints, there is an increasing demand for sophisticated, human-relevant testing systems that can mitigate the risks associated with ID-ADC candidates, clarify their mechanisms of action, and furnish predictive data to inform clinical translation. This section examines various next-generation models crucial for the thorough evaluation of infectious disease ID-ADCs.

### 9.1 Organ-on-chip models for niche-specific infectious biology

Microfluidic organ-on-chip (OoC) technologies signify a substantial progression, enabling the accurate replication of human organ functionality, encompassing regulation of fluid dynamics, mechanical characteristics, and cellular interactions. These systems possess significant potential for ID-ADC testing by accurately recreating the particular biological barriers and niches relevant to infection processes. A lung-on-a-chip model can replicate the alveolar environment by incorporating a bilayer of alveolar epithelial and endothelial cells exposed to cyclical mechanical stretching. This paradigm is essential for investigating ID-ADCs targeting pulmonary tuberculosis or *Pseudomonas aeruginosa* infection. This allows researchers to evaluate barrier permeability, specifically monitoring the capacity of an administered ID-ADC to traverse from the vascular channel into the alveolar airspace, where infected macrophages or biofilms are located (Yamazaki et al., 2021).

Moreover, including biosensors into these organ-on-chip systems facilitates real-time monitoring of essential parameters, including pH and reactive oxygen species (ROS), thereby confirming the activation of microenvironment-responsive linkers in the context of an infection. A liver-on-a-chip model that includes hepatocytes, Kupffer cells, and stellate cells can function as a distinctive platform to assess ID-ADCs aimed at hepatic-stage malaria hypnozoites or the amastigotes of Leishmania donovani. These sophisticated models are revolutionary, providing insights into ID-ADC biodistribution, target engagement, and activation that closely resemble human physiology (Chen et al., 2019). Representative advanced *in vitro* models and the specific ID-ADC metrics they are designed to evaluate are detailed in Table 9.

**TABLE 9:** Advanced organ-on-chip models for preclinical ADC screening.

Tissue interface	Pathogen application	ADC metric evaluated	Key advantage	References
Lung-on-a-Chip	*M. tuberculosis*, Influenza, RSV	Aerosolized delivery and penetration: ability of ADCs to cross the alveolar-capillary barrier	Simulates breathing-induced mechanical strain on infected cells	MacGregor et al. (2019), Riccardi et al. (2023)
Gut-on-a-Chip	*Salmonella*, *Vibrio cholerae*	Microbiome Preservation: Ensuring the ADC payload targets the pathogen without causing dysbiosis	Incorporates peristalsis and human commensal flora	Jain et al. (2024), Sasso et al. (2023)
Liver-on-a-Chip	Plasmodium (Malaria), Hepatitis B	Metabolic Stability and Clearance: Determining if the linker survives hepatic first-pass metabolism	Models the complex “Liver Stage” of parasites often neglected in 2D culture	Duvall et al. (2023)
Blood-Brain Barrier (BBB)	Trypanosoma (sleeping sickness), meningitis	Targeted transcytosis: efficacy of ADC antibodies designed to cross the BBB via transferrin receptors	Replaces failure rates of small molecules with targeted antibody transport	Doherty et al. (2025), Parakh et al. (2021), Pardridge (2023)
Granuloma-on-a-Chip	*Mycobacterium tuberculosis*	Deep Tissue Diffusion: Testing if ADCs can penetrate the necrotic, hypoxic center of a granuloma	Recreates the structural “fortress” that protects latent bacteria	Jin Y et al. (2022), Sen et al. (2023)

### 9.2 Three-Dimensional (3D) granuloma and biofilm models

Chronic infections are frequently marked by intricate three-dimensional formations like granulomas and biofilms, which operate as substantial physical and chemical obstructions that hinder drug infiltration. Developing three-dimensional human granuloma models using *Mycobacterium* TB-infected primary human macrophages embedded in collagen or fibrin matrix replicates the multicellular structure of tuberculosis lesions. In these models, the penetration kinetics of fluorescently labeled ID-ADCs can be visualized through confocal microscopy, enabling researchers to determine whether the conjugates can effectively diffuse into the hypoxic and necrotic core of the granuloma or if they are restricted to the more permeable peripheral layers (Bai et al., 2019).

*In vitro* biofilm models that replicate the shear stress and nutrition gradients of clinical situations, such as catheter-associated infections or cystic fibrosis-related lung infections, are essential for evaluating ID-ADC efficacy against biofilm-embedded bacteria. Assessing an anti-biofilm ID-ADC in these models offers insights on its capacity to infiltrate dense extracellular matrices and eradicate metabolically inactive bacteria, a difficulty often faced by traditional antibiotics (Ou et al., 2018). Integrating these 3D models into the ID-ADC testing procedure enables researchers to obtain crucial spatial pharmacokinetic data necessary for comprehending drug action in intricate infection microenvironments.

### 9.3 Humanized murine models for systemic and immune-responsive assessment

Traditional *in vitro* and *ex vivo* models frequently inadequately replicate systemic pharmacokinetics, biodistribution, and interactions with the human immune response in the pursuit of effective ID-ADCs for infectious illnesses. These elements are essential for comprehending the functionality of ID-ADCs in authentic biological situations. Consequently, *in vivo* systems, especially humanized mouse models, are becoming essential for these assessments.

Humanized mouse models, particularly NSG-SGM3 mice engrafted with human hematopoietic stem cells, exhibit a functionally human immune system comprising myeloid and lymphoid lineages. These models are especially proficient in assessing ID-ADCs that depend on human Fc effector activities, such as antibody-dependent cellular cytotoxicity (ADCC) or antibody-dependent cellular phagocytosis (ADCP), for the elimination of infected cells. ID-ADCs targeting HIV-infected cells or Leishmania-infected macrophages can only be accurately evaluated in hosts with the appropriate human Fcγ receptors on immune effector cells, facilitating a genuine assessment of immune-mediated pathogen clearance (Zhu F et al., 2023).

Furthermore, sophisticated models that incorporate both human liver tissue and a fully formed human immune system are being developed for critical illnesses, such as visceral leishmaniasis. These dual-humanization models yield essential insights into the organ-specific targeting of ID-ADCs and their related toxicities, establishing a more comprehensive framework for assessing the therapeutic efficacy and safety of novel ID-ADCs in the treatment of complex infections, such as those caused by Leishmania species (Choi W et al., 2020).

### 9.4 Advanced imaging and omics for mechanistic deconstruction

To obtain more refined insights beyond basic endpoint measures like pathogen burden reduction, sophisticated analytical methods must be employed to clarify the specific mechanisms of ID-ADC action at both molecular and cellular levels. Techniques such as spatial pharmacology, namely multiplexed ion beam imaging (MIBI) or imaging mass cytometry (IMC), facilitate the concurrent observation of many parameters on a single tissue section from infected mice. This comprehensive study may concurrently identify the ID-ADC, the target antigen, apoptotic markers in afflicted cells, a cleavage-activated fluorescent reporter for payload release, and diverse immune cell subsets (Kanamori et al., 2019).

High-dimensional spatial analyses produce intricate maps that elucidate both the operational areas of the ID-ADC and the particular cell populations it influences, along with the resultant consequences on the microenvironment. This is additionally enhanced by single-cell transcriptomics and proteomics (e.g., scRNA-seq, CyTOF), which can clarify the diverse cellular responses to treatment. This research delineates the precise populations, including infected macrophages and bystander T cells, that ingested the ID-ADC, identifies activated cell death or stress pathways, and evaluates potential larger modulations in immune signaling networks (Quinn et al., 2025).

The integration of spatial imaging and single-cell analytics transforms ID-ADC testing from a rudimentary efficacy assessment to a comprehensive mechanistic analysis of therapeutic effects and adverse reactions, facilitating more informed decision-making in the development and implementation of these targeted therapies.

## 10 Challenges and knowledge gaps

The translation of ID-ADCs from oncology to infectious diseases is limited by fundamental biological, chemical, regulatory, and economic differences between self-derived tumors and foreign, evolving pathogens. These constraints are structural rather than incremental and define the current boundaries of infectious disease ADC development.

### 10.1 Antigen discovery and accessibility

A major bottleneck is the scarcity of well-characterized, ID-ADC-suitable pathogen antigens. Many pathogens actively obscure surface targets through antigen masking, such as the glycolipid-rich envelopes of mycobacteria, or through rapid antigenic variation, as observed in malaria and trypanosomes (Johnson et al., 2022). For numerous neglected tropical diseases and antimicrobial-resistant organisms, comprehensive antigenic atlases and structurally validated surface targets remain unavailable, restricting ID-ADC development to a narrow set of empirically identified antigens.

### 10.2 Internalization and payload delivery barriers

Even when suitable antigens are identified, productive internalization is not guaranteed. Unlike oncology, receptor-mediated endocytosis is not universal in infectious diseases, as bacterial capsules and surface glycocalyxes frequently impede antibody-mediated uptake. Intracellular pathogens such as *Mycobacterium tuberculosis* and Leishmania spp. can further disrupt phagolysosomal maturation, limiting exposure to linker-cleaving conditions (Noy et al., 2018). Consequently, ID-ADCs may be misrouted to non-permissive compartments, resulting in insufficient payload release and impractically high dosing requirements (Aoyama et al., 2024).

### 10.3 Structural and biochemical knowledge gaps

Rational ID-ADC design is constrained by limited structural and biochemical data. High-resolution structures of pathogen-derived enzymes suitable for selective linker activation are available for only a small fraction of relevant targets (Aung et al., 2021). This limitation is compounded by the lack of quantitative *in vivo* measurements describing infectious microenvironments, including pH gradients in granulomas, redox conditions within parasitized phagosomes, and protease activity in bacterial biofilms, which are often inferred rather than directly measured (Arrigoni et al., 2023; Miwa et al., 2023).

### 10.4 The payload paradox

Payload selection presents a persistent trilemma between potency, selectivity, and conjugatability. Many conventional antimicrobials lose activity upon linker attachment, while emerging payload classes, including nucleotide analogues and metal-based complexes, frequently exhibit altered pharmacokinetics or aggregation when conjugated (Yamazaki et al., 2021). In particular, metallo-drug payloads are susceptible to coordination sphere disruption or trans-chelation during standard bioconjugation, potentially leading to inactivation or off-target toxicity (Hoffmann et al., 2020). Addressing this paradox requires payloads designed specifically for ID-ADC use, incorporating built-in conjugation handles and optimized physicochemical properties (Marone et al., 2025; Spangler et al., 2018).

### 10.5 Manufacturing and scale-up constraints

Current ADC manufacturing models, optimized for oncology, are poorly aligned with the economic realities of neglected tropical diseases and antimicrobial resistance, particularly in low- and middle-income countries (Tanaka et al., 2024). The lack of decentralized manufacturing infrastructure and sustainable business models represents a translational barrier comparable to scientific limitations (Cheng-Sánchez et al., 2022). Without innovations such as plant-based expression systems, continuous manufacturing, or supply-chain-resilient formulations, equitable deployment of ID-ADCs remains unlikely (Mckertish and Kayser, 2021).

### 10.6 Regulatory uncertainty

Regulatory frameworks for ADCs remain largely oncology-centric, providing limited guidance on acceptable safety margins, immunogenicity assessment, and efficacy endpoints for antimicrobial payloads (Chang et al., 2025; Petersen et al., 2024). Uncertainty regarding appropriate preclinical models, particularly for chronic versus acute infections, further increases development risk and cost (Theocharopoulos et al., 2020), discouraging investment and slowing translation of ID-ADCs (Bulger et al., 2023).

### 10.7 The translational valley of death

The convergence of scientific, manufacturing, and regulatory challenges culminates in a pronounced translational “valley of death,” especially for neglected tropical diseases. While academic studies often demonstrate promising ID-ADC proof-of-concept, advancement commonly stalls during lead optimization, pharmacokinetic and toxicological evaluation, or GMP process development due to limited resources and infrastructure (Theocharopoulos et al., 2021). As a result, many potentially transformative ID-ADC strategies remain confined to the literature (Chandrabatla, 2025).

The multifaceted translational barriers facing infectious disease ID-ADCs, alongside proposed interdisciplinary solutions, are synthesized in Table 10.

**TABLE 10:** Key translational challenges and proposed solutions for infectious disease ADCs.

Challenge category	Specific translational hurdle	Root cause/nature of the gap	Proposed solution/Enabling technology	References
1. Antigen discovery and validation	Lack of well-characterized, “ADC-suitable” pathogen surface antigens	Pathogen surfaces are often variable, masked, or non-protein	- High-throughput structural biology: Cryo-electron tomography (cryo-ET) of native pathogen surfaces	Cini et al. (2018), Wehrmüller et al. (2024)
			- AI-driven surfaceome mapping	
			- Open-access antigen databases	
	Antigenic variation (e.g., malaria, trypanosomes)	​	​	Long et al. (2025)
	Inefficient internalization of target-bound ADC.	​	​	Dong et al. (2018)
2. Payload design and delivery	The Payload Paradox: Balancing pathogen potency, host-cell selectivity, and chemical conjugatability	Most antimicrobials are not designed for conjugation; modification kills activity	- Development of pathogen-centric payload classes	Lee et al. (2023), Lepland et al. (2020), Šeborová et al. (2022)
			- “Smart” linker engineering to enhance intracellular trafficking	
	Inefficient release in the correct sub-cellular compartment	​	- Use of bispecific antibodies targeting both pathogen and host receptors to enhance delivery	Stoppa et al. (2024), Zaleski et al. (2024)
3. Preclinical modeling	Inadequate models that recapitulate human pathophysiology and ADC pharmacology	Standard models fail to mimic complex infection niches	- Adoption of advanced *in vitro* models, including organ-on-chip and 3D granuloma models	Chen et al. (2018), Govdi et al. (2022)
			- Humanized mouse models for immune evaluation	
	Lack of human immune components for evaluating immune-mediated functions	​	​	Baryakova et al. (2024)
4. Chemistry and conjugation	Instability or inactivation of novel payloads during bioconjugation	Standard conjugation can disrupt coordination spheres of metallo-drugs	- Development of bioorthogonal, site-specific conjugation methods	Chidkoksung et al. (2024), Piersimoni et al. (2021), Sograte-Idrissi et al. (2019)
	Poor solubility/aggregation of hydrophobic or charged payloads	​	- Design of tailored ligands for metallo-drugs to maintain solubility and activity	Beaumont et al. (2018), Liu et al. (2025)
5. Manufacturing and access	Prohibitive cost and complex supply chain for ADC production in LMICs	High-cost GMP processes, lack of economic incentives	- Innovative biomanufacturing platforms	Cengiz et al. (2021), Chigoho et al. (2021), Hervé-Aubert et al. (2018)
			- Establishment of Public-Private Partnerships (PPPs) to promote decentralized manufacturing	
	Lack of economic incentives in LMICs	​	- Development of thermostable formulations for reduced cold-chain reliance	Tatiparti et al. (2020), Yeo et al. (2024)
6. Regulatory and translational pathway	Regulatory uncertainty for non-oncology ADCs	Lack of clear regulatory guidance for antimicrobial payloads	- Proactive regulatory engagement with agencies (WHO, FDA) to tailor development pathways	Choi et al. (2024), Mapanao et al. (2018), Palacio-Castañeda et al. (2022)
	Funding gap from academic proof-of-concept to clinical candidate development	​	- Establishment of integrated translational consortia for collaborative development	Lim et al. (2023), Richardson et al. (2019)

## 11 Future directions and roadmap

The development of ID-ADCs for infectious diseases is at a pivotal juncture. While proof-of-concept studies demonstrate feasibility, translating ID-ADCs into effective therapeutics requires interdisciplinary approaches that integrate structural biology, computational modeling, and equitable translational strategies. This roadmap outlines strategic priorities for advancing ID-ADCs from concept to accessible therapies, emphasizing systems-level integration, metal-based payloads, multi-specific constructs, and AI-driven design pipelines. Collectively, these strategies illustrate how future ID-ADC development will integrate rational antibody, linker, and payload design with predictive modeling, pathogen-specific triggers, and scalable translational pipelines, ensuring that design principles guide all stages from discovery to deployment.

### 11.1 Digital and structural target discovery

A central challenge in infectious disease ID-ADC development is identifying suitable target antigens. Large-scale mapping of the “druggable surfaceome” of pathogens should combine experimental structural biology with computational predictions. High-throughput cryo-electron microscopy (cryo-EM) and tomography, coupled with surface biotinylation and proteomics, can catalog accessible epitopes (Kyeong et al., 2022; Pilarczyk et al., 2019). Integration with AI-based predictive tools like AlphaFold2 enables identification of conserved external loops suitable for ID-ADC targeting.

This approach should extend to advanced computational-experimental pipelines:

1. AI-Driven Antibody Design: Machine learning can forecast antibody-antigen interactions, enhancing selection and optimization for ID-ADCs (Mohamed et al., 2025).
2. Molecular Dynamics Simulations: In silico modeling of full ID-ADC structures evaluates stability and interactions in biological contexts (Hills et al., 2022).
3. Systems Pharmacology: Integrated PK/PD models predict ID-ADC distribution in complex infection environments, including granulomas and biofilms (Kim et al., 2018).
4. Coupled Validation: Organ-on-chip and 3D infection models provide experimental feedback that refines computational predictions (Adélaïde et al., 2023).
5. By combining structural mapping with AI and modeling, researchers can accelerate target identification, optimize ID-ADC design, and enhance the predictive power of preclinical studies (Majumder and Zhang, 2025; Rodríguez-Martínez et al., 2020).

### 11.2 Directed evolution of pathogen-responsive linker chemistry

Linker design must evolve beyond oncology-inspired motifs to exploit pathogen-specific characteristics. Directed evolution and combinatorial peptide libraries can identify highly specific cleavage sequences for pathogens such as *Mycobacterium tuberculosis* or multidrug-resistant bacteria (Cebriá-Mendoza et al., 2019).

Chemical linkers responsive to pathogen-specific signals—such as bacterial β-lactamases or parasite glycosidases—and “AND-gate” or “OR-gate” designs that require dual cues for payload release, can enhance selectivity and minimize off-target effects in ID-ADCs (Lavoie et al., 2021; Pires et al., 2021).

### 11.3 Metal-based ADC payloads

Metal-containing drugs offer unique mechanisms that bypass traditional antimicrobial resistance. Research priorities for ID-ADCs include:

1. Library Synthesis: Generating diverse libraries of inert, redox-active macrocyclic and phenanthroline-derived complexes for high-throughput screening against intracellular pathogens (Swain et al., 2021).
2. Bioorthogonal Conjugation: Developing chemistries that preserve metal-ligand coordination to maintain activity in ID-ADCs (Rossin and Robillard, 2021).
3. Kinetics and Stability Assays: *In vitro* studies replicating physiological conditions to evaluate ID-ADC payload release and stability (Zhang, 2025).
4. High-Throughput Screening: Identifying lead compounds with optimal selectivity, potency, and safety in host-cell models (Wang et al., 2021).
5. Mechanistic Elucidation: Structural and computational studies to clarify modes of action and guide rational ID-ADC design (Kuba et al., 2022; Taiariol et al., 2021).

### 11.4 Host-directed and multi-specific ADCs

Host-directed therapies can complement pathogen-targeting strategies by engaging receptors upregulated in infected cells or pathways critical for pathogen survival. Bispecific ADCs that simultaneously bind pathogen antigens and host receptors may improve internalization and payload precision (Heath et al., 2025; Kleinman et al., 2024).

Multi-specific ID-ADCs reduce risks of immune evasion and resistance, while synthetic biology and CRISPR-Cas9 can enable dynamic, context-responsive antibodies. Combining these strategies with immune checkpoint modulators may further enhance T-cell-mediated clearance (Lee et al., 2025; Silva et al., 2024).

### 11.5 Translational strategies and accessibility

To ensure global reach, particularly in LMICs, several translational priorities must be addressed:

1. Developability: ID-ADCs should utilize stable, high-expression antibodies, thermally resilient linkers, and cost-effective payloads (Alexander et al., 2024; Spring et al., 2024).
2. Public-Private Partnerships: Strategic collaborations among governments, NGOs, and industry enable infrastructure and funding for scalable ID-ADC production (Yang et al., 2024).
3. Innovative Manufacturing: Investment in LMIC-adapted platforms, such as plant-based or yeast expression systems, reduces production costs and accelerates output (Niles et al., 2018).
4. Regulatory Engagement: Continuous dialogue with agencies like WHO, FDA, and EMA streamlines preclinical and clinical development for ID-ADCs (Javaid et al., 2021; Kopp et al., 2022; Zhao et al., 2022).
5. Sustainability and Access: Supply chain optimization and equitable pricing ensure affordability and availability of ID-ADCs for populations most in need (Lu et al., 2025; Rudin et al., 2023).

The strategic progression necessary in all aspects of infectious disease ADC development, from discovery to deployment, is encapsulated in Table 11. This integrated pipeline delineates the transition from basic technologies to the sophisticated, egalitarian solutions required by 2030 and beyond for ID-ADCs.

**TABLE 11:** Integrated translational pipeline.

Focus area	Current state (Foundational)	Future frontier (2030+)	Role in translation	References
Antigen discovery	Genomic/Proteomic mapping	Direct Cryo-EM of native pathogens in tissues	Identifies “*In-Vivo*” stable targets	Yamazoe et al. (2025), Yoder et al. (2019)
Linker design	Single-trigger (Protease)	Multivariate “AND-gate” biosensing linkers	Maximizes precision/minimizes off-target effects	Aoyama et al. (2024), Lee et al. (2021)
Payload class	Repurposed cytotoxins/antibiotics	Catalytic Metallo-drugs and siRNA.	Overcomes antimicrobial resistance (AMR) and improves therapeutic efficacy	Balamkundu and Liu (2023a), Zhang B et al. (2024)
Preclinical models	2D Cell Culture/Basic Murine	Infection-on-Chip and Humanized Mice	Reduces clinical failure rates by closely modeling human pathophysiology	Chuprakov et al. (2021), Petersen et al. (2024), Watanabe et al. (2024)
Manufacturing	High-cost, cold-chain dependent	Cell-free, thermostable platforms	Enables global reach for neglected tropical diseases (NTDs) and improves supply chain efficiency	Aoyama et al. (2024), Shia et al. (2025), Usama et al. (2021)

## 12 Conclusion

Antibody-drug conjugates exemplify a model of precision medicine, with potential applications that extend well beyond the oncology domain in which they were initially developed. For infectious and neglected tropical diseases, hindered by medication toxicity, resistance, and inaccessible pathogen reservoirs, ID-ADCs present a revolutionary strategic framework: the precise delivery of potent therapeutic agents directly to the site of infection. This review has systematically outlined that translating this concept into successful medicines necessitates comprehensive re-engineering from foundational principles.

The fundamental structure of an ID-ADC cannot merely replicate targets from the oncology framework. It necessitates a profound synthesis of pathogen biology, structural understanding, and innovative chemistry. The antibody must be engineered to target conserved, internalizing epitopes on complex pathogen surfaces, guided by cryo-EM and computational modeling. The linker must transition from a stable tether to a biosensor, designed to cleave in response to distinctive chemical signatures—such as specific proteases, acidic pH, or oxidative stress—present in infectious microenvironments. The payload must evolve from cytotoxic agents that eliminate human cells to pathogen-specific antimicrobials, immunomodulators, or innovative chemotypes such as redox-active metallo-drugs, which possess distinct mechanisms capable of overcoming existing resistance.

The future remains fraught with obstacles, including antigen identification challenges, intracellular transport impediments, the complexity of metallo-drug conjugation, and the difficulty of large-scale manufacturing for global health deployment. Nevertheless, these gaps delineate a clear and compelling research agenda. Success depends on a cooperative, interdisciplinary approach that leverages contemporary computational frameworks for rational ID-ADC design, incorporates sophisticated organ-on-chip and 3D infection models for human-relevant testing, and establishes sustainable translational pathways.

The exploration of ID-ADCs for infectious disease treatment is only beginning. By grounding development in a chemistry-driven, structurally informed, and physiologically sophisticated methodology, the field can move beyond promising prototypes. The overarching objective is to establish a new class of precision anti-infectives—advanced molecular systems capable of identifying and eradicating infections within complex host environments. Achieving this goal will not only generate novel therapies for persistent diseases such as tuberculosis, drug-resistant malaria, and visceral leishmaniasis, but will also create a flexible platform adaptable to future pandemic threats. This study delineates the convergence of chemistry, biology, and translational science as the foundational framework for this undertaking.

## References

[B1] Abdalhamed A. M. Ghazy A. A. Zeedan G. S. G. (2021). Studies on multidrug-resistance bacteria in ruminants with special interest on antimicrobial resistances genes. Adv. Animal Veterinary Sci. 9 (6), 835–844. 10.17582/journal.aavs/2021/9.6.835.844

[B2] Abdel‐Rahman L. H. Alzarzah S. F. Abdel‐Hameed M. Shehata M. R. El‐Saghier A. M. M. (2024). A new bio‐active Schiff base ligand and its Ni(II), Cu(II), Ag(I), Zn(II), Cd(II), and La(III) binuclear complexes: synthesis, DFT analysis, antimicrobial, DNA interaction, COXII inhibition, and molecular docking studies. Appl. Organomet. Chem. 38 (9), e7631. 10.1002/aoc.7631

[B4] Adélaïde M. Salnikov E. S. Ramos‐Martín F. Aisenbrey C. Sarazin C. Bechinger B. (2023). The mechanism of action of SAAP-148 antimicrobial peptide as studied with NMR and molecular dynamics simulations. Pharmaceutics 15 (3), 761. 10.3390/pharmaceutics15030761 36986623 PMC10051583

[B5] Adhikari A. Chen I. A. (2025). Antibody‐Nanoparticle conjugates in therapy: combining the best of two worlds. Small 21 (15), e2409635. 10.1002/smll.202409635 40051146 PMC12001320

[B6] Ahmad F. J. (2022). Nanoparticles in the era of antimicrobial resistance. Pak. Biomed. J., 5, 01. 10.54393/pbmj.v5i12.837

[B7] Alexander S. Aleem U. Jacobs T. W. Frizziero M. Foy V. Hubner R. (2024). Antibody-drug conjugates and their potential in the treatment of patients with biliary tract cancer. Cancers 16 (19), 3345. 10.3390/cancers16193345 39409965 PMC11476249

[B8] Ali M. T. Rahman T. Palit P. Uddin M. I. Seidel V. (2025). Targeting the zinc metalloprotease gp63 of Leishmania for vaccine design and new drug discovery using immunoinformatics, molecular docking and molecular dynamics simulation studies. Exp. Parasitol. 277, 109009. 10.1016/j.exppara.2025.109009 40907690

[B9] Alradwan I. Alnefaie M. Fayez N. Aodah A. Majrashi M. Alturki M. (2025). Strategic and chemical advances in antibody–drug conjugates. Pharmaceutics 17 (9), 1164. 10.3390/pharmaceutics17091164 41012501 PMC12473541

[B10] Amaral L. Debona D. Costa L. Silva A. Oliveira J. Rodrigues F. (2019). Biochemical insights into basal and induced resistance in cabbage to black rot. J. Phytopathology 167 (7-8), 390–403. 10.1111/jph.12808

[B12] Aoyama M. Tada M. Yokoo H. Ito T. Misawa T. Demizu Y. (2024). Linker and conjugation site synergy in antibody–drug conjugates: impacts on biological activity. Bioconjugate Chem. 35 (10), 1568–1576. 10.1021/acs.bioconjchem.4c00348 39363433 PMC11488503

[B13] Arrigoni R. Santacroce L. Ballini A. Palese L. L. (2023). AI-Aided search for new HIV-1 protease ligands. Biomolecules 13 (5), 858. 10.3390/biom13050858 37238727 PMC10216636

[B14] Aung A. Cui A. Soleimany A. P. Bukenya M. Lee H. Cottrell C. A. (2021). Spatially regulated protease activity in lymph nodes renders B cell follicles a sanctuary for retention of intact antigens. 10.1101/2021.11.15.468669

[B15] Baah S. Laws M. Rahman K. M. (2021). Antibody–drug conjugates—A tutorial review. Molecules 26 (10), 2943. 10.3390/molecules26102943 34063364 PMC8156828

[B16] Baalmann M. Ziegler M. J. Werther P. Wilhelm J. Wombacher R. (2019). Enzymatic and site-specific ligation of minimal-size tetrazines and triazines to proteins for bioconjugation and live-cell imaging. Bioconjugate Chem. 30 (5), 1405–1414. 10.1021/acs.bioconjchem.9b00157 30883100

[B17] Bai C. Reid E. E. Wilhelm A. Shizuka M. Maloney E. K. Laleau R. (2019). Site-specific conjugation of the indolinobenzodiazepine dgn549 to antibodies affords antibody–drug conjugates with an improved therapeutic index as compared with lysine conjugation. Bioconjugate Chem. 31 (1), 93–103. 10.1021/acs.bioconjchem.9b00777 31747250

[B18] Balamkundu S. Liu C. (2023a). Lysosomal cleavable peptide linkers in antibody-drug conjugates. 10.20944/preprints202305.1084.v1 PMC10669454 38002080

[B19] Balamkundu S. Liu C. (2023b). Lysosomal-cleavable peptide linkers in antibody–drug conjugates. Biomedicines 11 (11), 3080. 10.3390/biomedicines11113080 38002080 PMC10669454

[B20] Barros D. P. d. Reed P. Alves M. M. Santos R. Oliva A. (2021). Biocompatibility and antimicrobial activity of nanostructured lipid carriers for topical applications are affected by type of oils used in their composition. Pharmaceutics 13 (11), 1950. 10.3390/pharmaceutics13111950 34834365 PMC8618763

[B21] Baryakova T. Hsu C. Segatori L. McHugh K. (2024). Novel approaches to label the surface of *S. aureus* with DBCO for click chemistry-mediated deposition of sensitive cargo. Bioconjug. Chem. 36 (6), 1157–1168. 10.1021/acs.bioconjchem.4c00575 40398634

[B22] Basarab G. S. Ghorpade S. R. Gibhard L. Mueller R. Njoroge M. Peton N. (2022). Spiropyrimidinetriones: a class of dna gyrase inhibitors with activity against mycobacterium tuberculosis and without cross-resistance to fluoroquinolones. Antimicrob. Agents Chemother. 66 (4), e0219221. 10.1128/aac.02192-21 35266826 PMC9017349

[B23] Beaumont M. Bacher M. Opietnik M. Gindl‐Altmutter W. Potthast A. Rosenau T. (2018). A general aqueous silanization protocol to introduce vinyl, mercapto or azido functionalities onto cellulose fibers and nanocelluloses. Molecules 23 (6), 1427. 10.3390/molecules23061427 29895798 PMC6100551

[B24] Benjamin S. R. Jackson C. P. Fang S. Carlson D. P. Guo Z. Tumey L. N. (2019). Thiolation of Q295: site-specific conjugation of hydrophobic payloads without the need for genetic engineering. Mol. Pharm. 16 (6), 2795–2807. 10.1021/acs.molpharmaceut.9b00323 31067063

[B25] Biji A. Khatun O. Swaraj S. Narayan R. Rajmani R. S. Sardar R. (2021). Identification of COVID-19 prognostic markers and therapeutic targets through meta-analysis and validation of omics data from nasopharyngeal samples. EBioMedicine 70, 103525. 10.1016/j.ebiom.2021.103525 34392148 PMC8358265

[B26] Boni V. Sharma M. Patnaik A. (2020). The resurgence of antibody drug conjugates in cancer therapeutics: novel targets and payloads. Am. Soc. Clin. Oncol. Educ. Book. 40, e58–e74. 10.1200/edbk_281107 32315240

[B27] Bossowska-Nowicka M. Mielcarska M. B. Romaniewicz M. Kaczmarek M. M. Gregorczyk-Zboroch K. P. Struzik J. (2019). Ectromelia virus suppresses expression of cathepsins and cystatins in conventional dendritic cells to efficiently execute the replication process. BMC Microbiol. 19 (1), 92. 10.1186/s12866-019-1471-1 31077130 PMC6509786

[B28] Braniewska A. Skorzynski M. Sas Z. Dlugolecka M. Marszałek I. Kurpiel D. (2024). A novel process for transcellular hemoglobin transport from macrophages to cancer cells. Cell Commun. Signal. 22 (1), 570. 10.1186/s12964-024-01929-8 39605056 PMC11603754

[B29] Bukowski K. Rogalska A. Marczak A. (2024). Folate receptor alpha—a secret weapon in ovarian cancer treatment? Int. J. Mol. Sci. 25 (22), 11927. 10.3390/ijms252211927 39595996 PMC11593442

[B30] Bulger P. G. Conlon D. A. Cink R. D. Fernandez‐Cerezo L. Zhang Q. Thirumalairajan S. (2023). Drug-linkers in antibody–drug conjugates: perspective on current industry practices. Org. Process Res. Dev. 27 (7), 1248–1257. 10.1021/acs.oprd.3c00136

[B31] Cai H. Yip V. Lee M. V. Wong S. Saad O. M. Ma S. (2020). Characterization of tissue distribution, catabolism, and elimination of an anti–staphylococcus aureus THIOMAB antibody-antibiotic conjugate in rats. Drug Metabolism Dispos. 48 (11), 1161–1168. 10.1124/dmd.120.000092 32839277

[B32] Castro M. Erber A. Arana B. Cota G. Denkinger C. Harrison N. (2024). Involving patients in drug development for neglected tropical diseases (ntds): a qualitative study exploring and incorporating preferences of patients with cutaneous leishmaniasis into target product profile development. Plos Neglected Trop. Dis. 18 (2), e0011975. 10.1371/journal.pntd.0011975 38381805 PMC10965092

[B33] Cavaco M. Castanho M. A. R. B. Neves V. (2022). The use of antibody-antibiotic conjugates to fight bacterial infections. Front. Microbiol. 13, 835677. 10.3389/fmicb.2022.835677 35330773 PMC8940529

[B34] Cazzaniga G. Zambra M. Bongiolo S. Prpić H. Fasola E. Arrigoni F. (2025). Comparative enzymatic and stability assays reveal GPLG as an effective cathepsin B cleavable linker for tumor-targeting drug conjugates. ACS Omega 10 (36), 41783–41798. 10.1021/acsomega.5c05758 40978411 PMC12444563

[B35] Cebriá-Mendoza M. Sanjuán R. Domingo‐Calap P. (2019). Directed evolution of a mycobacteriophage. Antibiotics 8 (2), 46. 10.3390/antibiotics8020046 31027152 PMC6627502

[B36] Cen X. Xu H. Zhu H. Deng H. Wang Z. Xu J. (2025). Abstract 5733: design and synthesis of the novel camptothecin analog mf-6 for application into site-specific antibody-drug conjugate. Cancer Res. 85 (8_Suppl. ment_1), 5733. 10.1158/1538-7445.am2025-5733

[B37] Cengiz B. Sanyal R. Sanyal A. (2021). Tailoring aqueous dispersibility and biofunctionalization of carbon nanotubes using maleimide-containing clickable polymers. ACS Appl. Polym. Mater. 3 (11), 5707–5716. 10.1021/acsapm.1c00977

[B38] Chandrabatla V. P. (2025). Analytical techniques for antibody-drug conjugates: comprehensive insights. ADC Rev./J. Antibody-Drug Conjugates. 10.14229/jadc.2025.07.15.001

[B39] Chang H. Le H. K. Liu S. Shah D. K. (2025). PK/PD of positively charged ADC in mice. Pharmaceutics 17 (3), 377. 10.3390/pharmaceutics17030377 40143040 PMC11944646

[B40] Chaundler C. S. P. Lu H. Fu R. Wang N. Lou H. Almeida G. S. (2023). Kinetics and efficacy of antibody drug conjugates in 3d tumour models. 10.1101/2023.02.14.528517

[B41] Chen F. Ma K. Madajewski B. Zhuang L. Zhang L. Rickert K. (2018). Ultrasmall targeted nanoparticles with engineered antibody fragments for imaging detection of HER2-overexpressing breast cancer. Nat. Commun. 9 (1), 4141. 10.1038/s41467-018-06271-5 30297810 PMC6175906

[B42] Chen T. Yang Y. Zhang Z. Fu C. Zhang Q. Williams J. D. (2019). Native reversed-phase liquid chromatography: a technique for lcms of intact antibody–drug conjugates. Anal. Chem. 91 (4), 2805–2812. 10.1021/acs.analchem.8b04699 30661356 PMC6727645

[B43] Chen B. Zheng X. Wu J. Chen G. Yu J. Xu Y. (2025). Antibody–drug conjugates in cancer therapy: current landscape, challenges, and future directions. Mol. Cancer 24 (1), 279. 10.1186/s12943-025-02489-2 41184856 PMC12581584

[B44] Cheng D. Li W. Wang L. Lin T. Poiani G. J. Wassef A. (2019). Pharmacokinetics, pharmacodynamics, and pkpd modeling of curcumin in regulating antioxidant and epigenetic gene expression in healthy human volunteers. Mol. Pharm. 16 (5), 1881–1889. 10.1021/acs.molpharmaceut.8b01246 30860383 PMC6710832

[B45] Cheng‐Sánchez I. Moya‐Utrera F. Porras-Alcalá C. López‐Romero J. M. Sarabia F. (2022). Antibody-drug conjugates containing payloads from marine origin. Mar. Drugs 20 (8), 494. 10.3390/md20080494 36005497 PMC9410405

[B46] Chidkoksung K. Parakasikron N. Nuanualsuwan S. Khantasup K. (2024). Development of a latex agglutination test based on VH antibody fragment for detection of Streptococcus suis serotype 2. PLOS ONE 19 (4), e0299691. 10.1371/journal.pone.0299691 38568909 PMC10990187

[B47] Chigoho D. Lecocq Q. Awad R. Breckpot K. Devoogdt N. Keyaerts M. (2021). Site-specific radiolabeling of a human PD-L1 nanobody via maleimide–cysteine chemistry. Pharmaceuticals 14 (6), 550. 10.3390/ph14060550 34201323 PMC8228271

[B48] Cho H. Shim M. K. Moon Y. Song S. Kim J. Choi J. (2022). Tumor-specific monomethyl Auristatin E (MMAE) prodrug nanoparticles for safe and effective chemotherapy. Pharmaceutics 14 (10), 2131. 10.3390/pharmaceutics14102131 36297566 PMC9609178

[B49] Choi Y. Choi Y. Hong S. (2024). Recent technological and intellectual property trends in antibody–drug conjugate research. Pharmaceutics 16 (2), 221. 10.3390/pharmaceutics16020221 38399275 PMC10892729

[B50] Choi P. J. Park T. Cooper E. Dragunow M. Denny W. A. Jose J. (2020). Heptamethine cyanine dye mediated drug delivery: hype or hope. Bioconjugate Chem. 31 (7), 1724–1739. 10.1021/acs.bioconjchem.0c00302 32530288

[B51] Choi W. Park R. Kim D. K. Shin Y. Cho Y. Lee H. S. (2020). Mertansine inhibits mrna expression and enzyme activities of cytochrome p450s and uridine 5′-diphospho-glucuronosyltransferases in human hepatocytes and liver microsomes. Pharmaceutics 12 (3), 220. 10.3390/pharmaceutics12030220 32131538 PMC7150891

[B52] Chuprakov S. Ogunkoya A. O. Barfield R. M. Bauzon M. Hickle C. Kim Y. C. (2021). Tandem-cleavage linkers improve the *in vivo* stability and tolerability of antibody–drug conjugates. Bioconjugate Chem. 32 (4), 746–754. 10.1021/acs.bioconjchem.1c00029 33689309

[B53] Cini E. Faltoni V. Petricci E. Taddei M. Salvini L. Giannini G. (2018). Antibody drug conjugates (ADCs) charged with HDAC inhibitor for targeted epigenetic modulation. Chem. Sci. 9 (31), 6490–6496. 10.1039/c7sc05266a 30288233 PMC6144071

[B54] Dannheim F. M. Walsh S. J. Orozco C. T. Hansen A. H. Bargh J. D. Jackson S. (2022). All-in-one disulfide bridging enables the generation of antibody conjugates with modular cargo loading. Chem. Sci. 13 (30), 8781–8790. 10.1039/d2sc02198f 35975158 PMC9350601

[B55] Das D. K. Govindan R. Nikić I. Krammer F. Lemke E. A. Munro J. B. (2018). Direct visualization of the conformational dynamics of single influenza hemagglutinin trimers. Cell 174 (4), 926–937.e12. 10.1016/j.cell.2018.05.050 29961575 PMC6086748

[B56] Derking R. Sanders R. W. (2021). Structure‐guided envelope trimer design in hiv‐1 vaccine development: a narrative review. J. Int. AIDS Soc. 24 (S7), e25797. 10.1002/jia2.25797 34806305 PMC8606863

[B58] Doherty C. Wilbanks B. Jain S. Pearson K. Bakken K. Burgenske D. (2025). *in vivo* selection of anti-glioblastoma dna aptamers in an orthotopic patient-derived xenograft model. Nar. Cancer 7 (1), zcaf005. 10.1093/narcan/zcaf005 39968526 PMC11833697

[B59] Döhrmann S. Levin J. Cole J. N. Borchardt A. Amundson K. Almaguer A. (2024). CD388: a universally protective Drug-Fc Conjugate that targets influenza virus neuraminidase. 10.1101/2024.06.04.597465 PMC11964917 40097766

[B60] Dong L. Li C. Locuson C. Chen S. Qian M. G. (2018). A two-step immunocapture LC/MS/MS assay for plasma stability and payload migration assessment of Cysteine–Maleimide-based antibody drug conjugates. Anal. Chem. 90 (10), 5989–5994. 10.1021/acs.analchem.8b00694 29688004

[B61] Durbin K. Phipps C. Liao X. (2018). Mechanistic modeling of antibody–drug conjugate internalization at the cellular level reveals inefficient processing steps. Mol. Cancer Ther. 17 (6), 1341–1351. 10.1158/1535-7163.mct-17-0672 29592884

[B62] Duvall J. R. Thomas J. D. Bukhalid R. A. Catcott K. C. Bentley K. W. Collins S. D. (2023). Discovery and optimization of a sting agonist platform for application in antibody drug conjugates. J. Med. Chem. 66 (15), 10715–10733. 10.1021/acs.jmedchem.3c00907 37486969 PMC10424177

[B63] D’Agostino M. Innorcia S. Boccadoro M. Bringhen S. (2020). Monoclonal antibodies to treat multiple myeloma: a dream come true. Int. J. Mol. Sci. 21 (21), 8192. 10.3390/ijms21218192 33139668 PMC7662679

[B64] Erhardt E. Ursino M. Biewenga J. Jacobs T. Gasparini M. (2018). Bayesian knowledge integration for an in Vitro–in vivo correlation model. Biometrical J. 61 (5), 1104–1119. 10.1002/bimj.201700263 30259557

[B65] Falck G. Müller K. M. (2018). Enzyme-based labeling strategies for antibody–drug conjugates and antibody mimetics. Antibodies 7 (1), 4. 10.3390/antib7010004 31544857 PMC6698867

[B66] Ferdous S. Martin A. C. (2018). AbDb: antibody structure database-a database of PDB-derived antibody structures. Database, Abdb Antibody Structure Database—A Database Pdb-Derived Antibody Structures. 2018, 1–9. 10.1093/database/bay040 29718130 PMC5925428

[B67] Fidler M. Wilkins J. Hooijmaijers R. Post T. M. Schoemaker R. C. Trame M. N. (2019). Nonlinear mixed‐effects model development and simulation using nlmixr and related r open‐source packages. CPT Pharmacometrics Syst. Pharmacol. 8 (9), 621–633. 10.1002/psp4.12445 31207186 PMC6765694

[B68] Fu Z. Li S. Han S. Chen S. Zhang Y. (2022). Antibody drug conjugate: the “biological missile” for targeted cancer therapy. Signal Transduct. Target. Ther. 7 (1), 93. 10.1038/s41392-022-00947-7 35318309 PMC8941077

[B69] Fujita Y. Ishiwada N. Takei H. Suwabe S. Yarita K. Ohkusu M. (2019). Usefulness of gastric aspirate culture for diagnosing congenital immunodeficiency in an infant with Fungal pneumonia caused by Rasamsonia piperina. Tohoku J. Exp. Med. 247 (4), 265–269. 10.1620/tjem.247.265 31006737

[B70] Galkin A. Y. Gorshunov Y. V. Besarab O. B. Shchurska K. (2018). Biotechnology for obtaining hybrid positive control samples for immunoassay for detecting antibodies against *Chlamydia trachomatis* . Regul. Mech. Biosyst. 9 (2), 141–147. 10.15421/021821

[B71] Gao W. Chen Y. Zhang Y. Zhang Q. Zhang L. (2018). Nanoparticle-based local antimicrobial drug delivery. Adv. Drug Deliv. Rev. 127, 46–57. 10.1016/j.addr.2017.09.015 28939377 PMC5860926

[B72] Gao Y. Xia Y. Chen Y. Zhou S. Fang Y. Yu J. (2025). Key considerations based on pharmacokinetic/pharmacodynamic in the design of antibody-drug conjugates. Front. Oncol. 14, 1459368. 10.3389/fonc.2024.1459368 39850824 PMC11754052

[B73] García‐Alonso S. Ocaña A. Pandiella A. (2018). Resistance to antibody–drug conjugates. Cancer Res. 78 (9), 2159–2165. 10.1158/0008-5472.can-17-3671 29653942

[B74] Gautam I. Yarava J. Xu Y. Li R. Scott F. Mentink‐Vigier F. (2024). Comparative analysis of polysaccharide and cell Wall structure inAspergillus nidulansandAspergillus fumigatusby solid-state NMR. 10.1101/2024.08.13.607833 PMC11576540 39562136

[B76] Ghorbani M. Brooks B. R. Klauda J. B. (2020). Exploring dynamics and network analysis of spike glycoprotein of sars-cov-2. 10.1101/2020.09.28.317206 PMC7939993 33705760

[B77] Giese M. Davis P. D. Woodman N. Hermanson G. T. Pokora A. Vermillion M. (2021). Linker architectures as steric auxiliaries for altering enzyme-mediated payload release from bioconjugates. Bioconjugate Chem. 32 (10), 2257–2267. 10.1021/acs.bioconjchem.1c00429 34587447

[B78] Gingrich J. (2020). How the next generation antibody drug conjugates expands beyond cytotoxic payloads for cancer therapy. ADC Rev./J. Antibody-Drug Conjugates. 10.14229/jadc.2020.04.07.001

[B79] Gonen T. (2025). Cryoem milestones and future directions. Struct. Dyn. 12 (5_Suppl. ment), A256. 10.1063/4.0001045

[B80] Govdi A. I. Tokareva P. A. Rumyantsev A. M. Panov M. S. Stellmacher J. Alexiev U. (2022). 4,5-Bis(arylethynyl)-1,2,3-triazoles—A new class of fluorescent labels: synthesis and applications. Molecules 27 (10), 3191. 10.3390/molecules27103191 35630673 PMC9147796

[B81] Grant O. C. Montgomery D. W. Ito K. Woods R. J. (2020). Analysis of the sars-cov-2 spike protein glycan shield: implications for immune recognition. 10.1101/2020.04.07.030445 PMC7490396 32929138

[B82] Graziani E. Sung M. Ma D. Narayanan B. Marquette K. Puthenveetil S. (2020). Pf-06804103, a site-specific anti-her2 antibody–drug conjugate for the treatment of her2-expressing breast, gastric, and lung cancers. Mol. Cancer Ther. 19 (10), 2068–2078. 10.1158/1535-7163.mct-20-0237 32747418

[B83] Grunst M. W. Ladd R. A. Clark N. M. Gil H. M. Klenchin V. A. Mason R. D. (2023). Antibody-dependent cellular cytotoxicity, infected cell binding and neutralization by antibodies to the SIV envelope glycoprotein. PLOS Pathog. 19 (5), e1011407. 10.1371/journal.ppat.1011407 37253062 PMC10256149

[B84] Gui X. Deng M. Song H. Chen Y. Xie J. Li Z. (2019). Disrupting lilrb4/apoe interaction by an efficacious humanized antibody reverses t-cell suppression and blocks aml development. Cancer Immunol. Res. 7 (8), 1244–1257. 10.1158/2326-6066.cir-19-0036 31213474 PMC6677629

[B85] Guo C. Zhang H. Xie X. Liu Y. Sun L. Li H. (2018). H1N1 influenza virus epitopes classified by monoclonal antibodies. Exp. Ther. Med. 16 (3), 2001–2007. 10.3892/etm.2018.6429 30186431 PMC6122413

[B86] Guo P. Ma L. Lu Y. Dai Y. Yang T. Yang Y. (2023). Therapeutic targeting tongue squamous cell carcinoma via ICAM1 antibody-drug conjugates in preclinical models. 10.21203/rs.3.rs-3353522/v1

[B87] Guthmiller J. J. Han J. Utset H. A. Li L. Lan L. Y. Henry C. (2021). A public broadly neutralizing antibody class targets a membrane-proximal anchor epitope of influenza virus hemagglutinin. 10.1101/2021.02.25.432905

[B88] Hafeez U. Parakh S. Gan H. Scott A. M. (2020). Antibody–drug conjugates for cancer therapy. Molecules 25 (20), 4764. 10.3390/molecules25204764 33081383 PMC7587605

[B90] Heath B. Kaira B. G. Thakker D. Mohammed O. J. Choudhury R. Dave F. (2025). SC134-deruxtecan a fucosyl-GM1 targeting ADC for small cell lung cancer therapy. 10.21203/rs.3.rs-6888650/v1 PMC12366213 40830881

[B91] Hervé-Aubert K. Allard-Vannier É. Joubert N. Lakhrif Z. Alric C. Martin C. (2018). Impact of site-specific conjugation of ScFv to multifunctional nanomedicines using second generation maleimide. Bioconjugate Chem. 29 (5), 1553–1559. 10.1021/acs.bioconjchem.8b00091 29553717

[B92] Hills O. J. Yong C. W. Scott A. DeVine D. Smith J. M. Chappell H. F. (2022). Atomic-scale interactions between quorum sensing autoinducer molecules and the mucoid *P. aeruginosa* exopolysaccharide matrix. Sci. Rep. 12 (1), 7724. 10.1038/s41598-022-11499-9 35545629 PMC9095684

[B93] Hilst Q. v. Vasdev R. A. S. Preston D. Findlay J. A. Scottwell S. Ø. Giles G. I. (2019). Synthesis, characterisation and antimicrobial studies of some 2,6‐bis(1,2,3‐Triazol‐4‐yl)Pyridine Ruthenium(II) “click” complexes. Asian J. Org. Chem. 8 (4), 496–505. 10.1002/ajoc.201900088

[B94] Hoffmann R. M. Mele S. Cheung A. Larcombe-Young D. Bucaite G. Sachouli E. (2020). Rapid conjugation of antibodies to toxins to select candidates for the development of anticancer antibody-drug conjugates (ADCs). Sci. Rep. 10 (1), 8869. 10.1038/s41598-020-65860-x 32483228 PMC7264231

[B95] Homer J. A. Johnson R. Koelln R. A. Moorhouse A. D. Moses J. E. (2024). Strategic re-engineering of antibiotics. Nat. Rev. Bioeng. 3 (3), 213–229. 10.1038/s44222-024-00250-w 40384761 PMC12083850

[B96] Huang H. Lee W. Zou H. Li H. Zhang S. Li H. (2023). Antimicrobial peptides in Dendrobium officinale: genomic parameters, peptide structures, and gene expression patterns. Plant Genome 16 (3), e20348. 10.1002/tpg2.20348 37194434 PMC12807342

[B97] Inoue T. Shinnakasu R. Chie K. Yamamoto H. Sakakibara S. Ono C. (2022). Antibody feedback contributes to facilitating the development of omicron-reactive memory b cells in sars-cov-2 mrna vaccinees. J. Exp. Med. 220 (2), 1–12. 10.1084/jem.20221786 PMC9750191 36512034

[B98] Iwamoto S. Mori Y. Yamashita T. Ojima K. Akita K. Togano S. (2023). Trophoblast cell surface antigen-2 phosphorylation triggered by binding of galectin-3 drives metastasis through down-regulation of E-cadherin. J. Biol. Chem. 299 (8), 104971. 10.1016/j.jbc.2023.104971 37380081 PMC10392139

[B99] Jaafar A. Fix‐Tailler A. Mansour N. Allain M. Shebaby W. N. Faour W. H. (2020). Synthesis, characterization, antifungal and antibacterial activities evaluation of copper (II), zinc (II) and cadmium (II) chloride and bromide complexes with new (E)‐1‐(3,4‐dimethoxybenzylidene)‐4‐methylthiosemicarbazone ligand. Appl. Organomet. Chem. 34 (12), e5988. 10.1002/aoc.5988

[B100] Jadhav K. Abhang A. Kole E. Gadade D. Dusane A. Iyer A. (2025). Peptide–drug conjugates as next-generation therapeutics: exploring the potential and clinical progress. Bioengineering 12 (5), 481. 10.3390/bioengineering12050481 40428099 PMC12108627

[B101] Jain S. Griffith J. I. Porath K. A. Rathi S. Le J. Pasa T. I. (2024). Bystander effects, pharmacokinetics, and linker-payload stability of EGFR-targeting antibody-drug conjugates losatuxizumab vedotin and Depatux-M in glioblastoma models. Clin. Cancer Res. 30 (15), 3287–3297. 10.1158/1078-0432.ccr-24-0426 38743766 PMC11292202

[B102] Javaid F. Pilotti C. Camilli C. Kallenberg D. Bahou C. Blackburn J. W. D. (2021). Leucine-rich alpha-2-glycoprotein 1 (LRG1) as a novel ADC target. RSC Chem. Biol. 2 (4), 1206–1220. 10.1039/d1cb00104c 34458833 PMC8341842

[B103] Jiang Y. Xu X. Fan D. Liu P. Zhou M. Cheng M. (2024). Advancing tumor-targeted chemo-immunotherapy: development of the car-m-derived exosome-drug conjugate. J. Med. Chem. 67 (16), 13959–13974. 10.1021/acs.jmedchem.4c00753 39041307

[B104] Jiang X. Nabil W. N. N. Ze Y. Dai R. Xi Z. Xu H. (2024). Unlocking natural potential: antibody-drug conjugates with naturally derived payloads for cancer therapy. Phytotherapy Res. 39 (2), 789–874. 10.1002/ptr.8407 39688127

[B106] Jin Y. Zakeri S. E. Bahal R. Wiemer A. J. (2022). New technologies bloom together for bettering cancer drug conjugates. Pharmacol. Rev. 74 (3), 680–713. 10.1124/pharmrev.121.000499 35710136 PMC9553120

[B107] Johannes S. Sommer A. Lerchen H. (2021). Protease-sensitive linkers. Chem. Linkers Antibody–Drug Conjugates (ADCs), 173–212. 10.1039/9781839165153-00173

[B108] John S. Chen H. Deng M. Gui X. Wu G. Chen W. (2018). A novel anti-lilrb4 car-t cell for the treatment of monocytic aml. Mol. Ther. 26 (10), 2487–2495. 10.1016/j.ymthe.2018.08.001 30131301 PMC6171100

[B109] Johnson K. D. Delaney J. C. Guillard T. Reffuveille F. Varin J. Li K. (2022). Development of an antibody fused with an antimicrobial peptide targetingPseudomonas aeruginosa:a new approach to prevent and treat bacterial infections. 10.1101/2022.12.28.522163 PMC10508631 37676873

[B110] Kanamori T. Iwata Y. Segawa H. Yamamuro T. Kuwayama K. Tsujikawa K. (2019). Metabolism of butyrylfentanyl in fresh human hepatocytes: chemical synthesis of authentic metabolite standards for definitive identification. Biol. Pharm. Bull. 42 (4), 623–630. 10.1248/bpb.b18-00765 30930421

[B111] Khongorzul P. Ling C. Khan F. U. Ihsan A. U. Zhang J. (2020). Antibody–drug conjugates: a comprehensive review. Mol. Cancer Res. 18 (1), 3–19. 10.1158/1541-7786.mcr-19-0582 31659006

[B112] Kim R. Kanamaru S. Mikawa T. Prévost C. Ishii K. Ito K. (2018). RecA requires two molecules of Mg2+ ions for its optimal strand exchange activity *in vitro* . Nucleic Acids Res. 46 (5), 2548–2559. 10.1093/nar/gky048 29390145 PMC5861410

[B114] Kleinman D. Iqbal S. Ghosh A. K. Ogle S. D. Kaja S. Mitchnick M. (2024). PLL-g-PEG polymer inhibits antibody-drug conjugate uptake into human corneal epithelial cells *in vitro* . J. Ocular Pharmacol. Ther. 40 (7), 419–427. 10.1089/jop.2024.0019 38935528 PMC11564684

[B115] Kopp A. Hofsess S. Cardillo T. M. Govindan S. V. Donnell J. Thurber G. M. (2022). Antibody–drug conjugate Sacituzumab Govitecan drives efficient tissue penetration and rapid intracellular drug release. Mol. Cancer Ther. 22 (1), 102–111. 10.1158/1535-7163.mct-22-0375 36190986 PMC9812893

[B116] Krokhotin A. Du H. Hirabayashi K. Popov K. I. Kurokawa T. Wan X. (2019). Computationally guided design of single-chain variable fragment improves specificity of chimeric antigen receptors. Mol. Ther. - Oncolytics 15, 30–37. 10.1016/j.omto.2019.08.008 31650023 PMC6804740

[B117] Kuba W. Sohr B. Keppel P. Svatunek D. Humhal V. Stöger B. (2022). Oxidative desymmetrization enables the concise synthesis of a trans‐cyclooctene linker for bioorthogonal bond cleavage. Chem. – A Eur. J. 29 (3), e202203069. 10.1002/chem.202203069 36250260 PMC10098836

[B118] Kwon S. (2021). Market trend and current status of the research and development of antibody-drug conjugates. Biomed. Sci. Lett. 27 (3), 121–133. 10.15616/bsl.2021.27.3.121

[B119] Kwon Y. D. Chuang G. Zhang B. Bailer R. T. Doria‐Rose N. A. Gindin T. (2018). Surface-matrix screening identifies semi-specific interactions that improve potency of a near pan-reactive hiv-1-neutralizing antibody. Cell Rep. 22 (7), 1798–1809. 10.1016/j.celrep.2018.01.023 29444432 PMC5889116

[B120] Kyeong H. Cheon S. Kim H. Lee K. Ryu H. S. Han D. (2022). Discovery of proteins responsible for resistance to three chemotherapy drugs in breast cancer cells using proteomics and bioinformatics analysis. Molecules 27 (6), 1762. 10.3390/molecules27061762 35335125 PMC8954867

[B121] Labant M. (2024). Bioconjugates meet and promise to exceed expectations. Genet. Eng. Biotechnol. News 44 (3), 28–31. 10.1089/gen.44.03.12

[B122] Lavender H. Barendt T. A. Lucy D. Tang C. M. (2025). An antibody-drug conjugate exploiting a bacterial immune evasion mechanism is effective against multidrug resistant neisseria gonorrhoeae. 10.1101/2025.04.16.644158

[B123] Lavoie R. R. Gargollo P. C. Ahmed M. E. Kim Y. Baer E. Phelps D. A. (2021). Surfaceome profiling of rhabdomyosarcoma reveals B7-H3 as a mediator of immune evasion. Cancers 13 (18), 4528. 10.3390/cancers13184528 34572755 PMC8466404

[B124] Lee W. S. Prévost J. Richard J. Sluis R. M. v. d. Lewin S. R. Pazgier M. (2019). CD4-and time-dependent susceptibility of HIV-1-Infected cells to antibody-dependent cellular cytotoxicity. J. Virology 93 (10). 10.1128/jvi.01901-18 30842324 PMC6498039

[B125] Lee B. i. Park S. Park Y. Shin S. Choi J. Park M. (2021). Assessments of the *in vitro* and *in vivo* linker stability and catabolic fate for the ortho hydroxy-protected aryl sulfate linker by immuno-affinity capture liquid chromatography quadrupole time-of-flight mass spectrometric assay. Pharmaceutics 13 (1), 125. 10.3390/pharmaceutics13010125 33478046 PMC7836004

[B126] Lee T. Kim J. H. Kwon S. J. Seo J. Park S. H. Kim J. (2022). Site-selective antibody–drug conjugation by a proximity-driven s to n acyl transfer reaction on a therapeutic antibody. J. Med. Chem. 65 (7), 5751–5759. 10.1021/acs.jmedchem.2c00084 35319890

[B127] Lee J. Choi J. Kim E. Choi J. Kim S. Yang Y. (2023). Design of siRNA bioconjugates for efficient control of cancer-associated membrane receptors. ACS Omega 8 (39), 36435–36448. 10.1021/acsomega.3c05395 37810687 PMC10552107

[B128] Lee H. M. Abdul-Hadi K. Appleman V. A. Cardin D. Dong L. England D. (2025). Identification of a novel linker enabling the bioconjugation of a cyclic dinucleotide for the STING antibody-drug conjugate TAK-500. Bioconjugate Chem. 36 (11), 2423–2435. 10.1021/acs.bioconjchem.5c00424 41143331 PMC12635970

[B129] Lepland A. Asciutto E. K. Malfanti A. Simón-Gracia L. Sidorenko V. Vicent M. J. (2020). Targeting pro-tumoral macrophages in early primary and metastatic breast tumors with the CD206-Binding mUNO peptide. Mol. Pharm. 17 (7), 2518–2531. 10.1021/acs.molpharmaceut.0c00226 32421341

[B130] Li N. Li Z. Fu Y. Cao S. (2020). Cryo-em studies of virus-antibody immune complexes. Virol. Sin. 35 (1), 1–13. 10.1007/s12250-019-00190-5 31916022 PMC7035235

[B131] Liao Y. Luo D. Peng K. Zeng Y. (2021). Cyclophilin a: a key player for etiological agent infection. Appl. Microbiol. Biotechnol. 105 (4), 1365–1377. 10.1007/s00253-021-11115-2 33492451 PMC7829623

[B133] Lim J. Park M. Park Y. Park S. Lee J. Hwang S. (2023). Evaluation of *in vivo* prepared albumin-drug conjugate using immunoprecipitation linked LC-MS assay and its application to mouse pharmacokinetic study. Molecules 28 (7), 3223. 10.3390/molecules28073223 37049985 PMC10096712

[B134] Lindesmith L. C. Mallory M. L. Debbink K. Donaldson E. Brewer-Jensen P. D. Swann E. W. (2018). Conformational occlusion of blockade antibody epitopes, a novel mechanism of gii.4 human norovirus immune evasion. mSphere 3 (1). 10.1128/msphere.00518-17 29435493 PMC5806210

[B136] Liu Y. Jia Y. Yang K. Wang Z. (2020). Heterogeneous strategies to eliminate intracellular bacterial pathogens. Front. Microbiol. 11, 563. 10.3389/fmicb.2020.00563 32390959 PMC7192003

[B137] Liu X. Balligand T. Gall C. Ploegh H. L. (2025). A monoclonal anti-hemagglutinin stem antibody modified with zanamivir protects against both influenza A and B viruses. Proc. Natl. Acad. Sci. 122 (15), e2424889122. 10.1073/pnas.2424889122 40193611 PMC12012527

[B138] Llamazares C. Olmo N. S. d. Ortega P. Gómez R. Soliveri J. Mata F. J. d. l. (2019). Antibacterial effect of carbosilane metallodendrimers in planktonic cells of gram-positive and gram-negative bacteria and *Staphylococcus aureus* biofilm. Biomolecules 9 (9), 405. 10.3390/biom9090405 31450779 PMC6769849

[B139] Long R. Zuo H. Tang G. Zhang C. Yue X. Yang J. (2025). Antibody-drug conjugates in cancer therapy: applications and future advances. Front. Immunol. 16, 1516419. 10.3389/fimmu.2025.1516419 40469310 PMC12133739

[B141] Lovey A. Krel M. Borchardt A. Brady T. Cole J. N. Do Q. (2021). Development of novel immunoprophylactic agents against multidrug-resistant gram-negative bacterial infections. Antimicrob. Agents Chemother. 65 (11), e0098521. 10.1128/aac.00985-21 34370589 PMC8522721

[B142] Lu Y. Yang L. Zhang W. Li J. Peng X. Qin Z. (2022). Pharmacokinetics and pharmacodynamics of isopropoxy benzene guanidine against clostridium perfringens in an intestinal infection model. Front. Veterinary Sci. 9, 1004248. 10.3389/fvets.2022.1004248 36246309 PMC9557049

[B143] Lu M. Zeng Y. Hang J. Shi W. Huang W. Tang F. (2025). Direct preparation of site‐specific antibody–drug conjugates with unpurified antibodies in culture medium. ChemBioChem 26 (11), e202401082. 10.1002/cbic.202401082 40151029

[B144] Lucas A. Moody A. Schorzman A. Zamboni W. (2021). Importance and considerations of antibody engineering in antibody-drug conjugates development from a clinical pharmacologist’s perspective. Antibodies 10 (3), 30. 10.3390/antib10030030 34449544 PMC8395454

[B145] MacGregor P. González-Muñoz A. L. Jobe F. Taylor M. C. Rust S. Sandercock A. M. (2019). A single dose of antibody-drug conjugate cures a stage 1 model of African trypanosomiasis. PLOS Neglected Trop. Dis. 13 (5), e0007373. 10.1371/journal.pntd.0007373 31120889 PMC6532856

[B146] Maciel T. R. Funguetto-Ribeiro A. C. Olivo L. B. Teixeira F. E. G. Pacheco C. O. Araújo B. V. d. (2024). Improved malaria therapy with cationic nanocapsules demonstrated in plasmodium berghei-infected rodents using whole blood surrogate population pk/pd modeling. Pharmaceutics 16 (11), 1369. 10.3390/pharmaceutics16111369 39598493 PMC11597719

[B304] Majumder U. Zhu X. Custar D. Li D. Fangs H. McGonigle S. (2024). A novel concept for cleavable linkers applicable to conjugation chemistry – design, synthesis and characterization. ChemBioChem 26 (4). 10.1002/cbic.202400826 39424599

[B147] Majumder P. Zhang P. (2025). *In situ* cryo-electron microscopy and tomography of cellular and organismal samples. Curr. Opin. Struct. Biol. 93, 103076. 10.1016/j.sbi.2025.103076 40472449 PMC12374797

[B148] Mapanao A. K. Santi M. Faraci P. Cappello V. Cassano D. Voliani V. (2018). Endogenously triggerable ultrasmall-in-nano architectures: targeting assessment on 3D pancreatic carcinoma spheroids. ACS Omega 3 (9), 11796–11801. 10.1021/acsomega.8b01719 30320273 PMC6173554

[B149] Marei H. E. Cenciarelli C. Hasan A. (2022). Potential of antibody–drug conjugates (adcs) for cancer therapy. Cancer Cell Int. 22 (1), 255. 10.1186/s12935-022-02679-8 35964048 PMC9375290

[B150] Market M. Tennakoon G. Scaffidi M. Cook D. P. Angka L. Ng J. (2022). Preventing surgery-induced nk cell dysfunction using anti-tgf-β immunotherapeutics. Int. J. Mol. Sci. 23 (23), 14608. 10.3390/ijms232314608 36498937 PMC9737532

[B151] Marone R. Asllanaj E. Capoferri G. Schwede T. Jeker L. T. Lepore R. (2025). Impact of human genetic variation underlying resistance to antigen-specific immunotherapy. 10.1101/2025.05.02.651174

[B154] Matveev A. Krylov V. Khlusevich Y. Baykov I. Yashunsky D. Emelyanova L. (2019). Novel mouse monoclonal antibodies specifically recognizing β-(1→3)-D-glucan antigen. Plos One 14 (4), e0215535. 10.1371/journal.pone.0215535 31022215 PMC6483564

[B155] Mayer R. L. Impens F. (2021). Immunopeptidomics for next-generation bacterial vaccine development. Trends Microbiol. 29 (11), 1034–1045. 10.1016/j.tim.2021.04.010 34030969

[B158] Meng X. Sun W. Weng W. Shi J. Ma B. DeMarco K. D. (2024). Antibody-mediated co-delivery of programmable drug combinations. 10.21203/rs.3.rs-5181233/v1

[B159] Mitra A. K. (2019). Visualization of biological macromolecules at near-atomic resolution: cryo-electron microscopy comes of age. Acta Crystallogr. Sect. F. Struct. Biol. Commun. 75 (1), 3–11. 10.1107/s2053230x18015133 30605120 PMC6317457

[B160] Mitran C. J. Mena A. Gnidehou S. Banman S. L. Arango E. Lima B. A. S. (2019). Antibodies to cryptic epitopes in distant homologues underpin a mechanism of heterologous immunity betweenplasmodium vivaxpvdbp andplasmodium falciparumvar2csa. mBio 10 (5). 10.1128/mbio.02343-19 31594821 PMC6786876

[B161] Miwa K. Guo Y. Hata M. Hirano Y. Yamamoto N. Hoshino T. (2023). In silicoandlt;/iandgt; identification of inhibitory compounds for SARS-Cov-2 papain-like protease. Chem. Pharm. Bull. 71 (12), 897–905. 10.1248/cpb.c23-00622 38044142

[B162] Mohamed M. S. Elsaman T. Mohamed M. A. Eltayib E. M. Abdalla A. E. Idriss M. T. (2025). Identification of bacterial oligopeptidase B inhibitors from microbial natural products: molecular insights, docking studies, MD simulations, and ADMET predictions. Pharmaceuticals 18 (5), 709. 10.3390/ph18050709 40430528 PMC12114661

[B163] Mondal D. Ford J. W. Pinney K. G. (2018). Improved methodology for the synthesis of a cathepsin b cleavable dipeptide linker, widely used in antibody-drug conjugate research. Tetrahedron Lett. 59 (40), 3594–3599. 10.1016/j.tetlet.2018.08.021 31156276 PMC6541422

[B164] Mookherjee N. Anderson M. A. Haagsman H. P. Davidson D. J. (2020). Antimicrobial host defence peptides: functions and clinical potential. Nat. Rev. Drug Discov. 19 (5), 311–332. 10.1038/s41573-019-0058-8 32107480

[B165] Motley M. P. Banerjee K. Fries B. C. (2019). Monoclonal antibody-based therapies for bacterial infections. Curr. Opin. Infect. Dis. 32 (3), 210–216. 10.1097/qco.0000000000000539 30950853 PMC7050834

[B166] Mugenyi N. (2025). Global trends, drivers, and public health impacts of antimicrobial resistance: a comprehensive review of “a silent pandemic”. (preprint). 10.2196/preprints.78958

[B167] Mukherjee A. Waters A. Babić I. Nurmemmedov E. Glassy M. Kesari S. (2018). Antibody drug conjugates: progress, pitfalls, and promises. Hum. Antibodies 27 (1), 53–62. 10.3233/hab-180348 30223393

[B168] Muppa L. Velmani V. Nandhani N. B V. S. S. Radhakrishnan S. M B. A. (2024). Extended spectrum β-lactamase: tackling antibiotic resistance and overcoming treatment challenges. Int. J. Curr. Sci. Res. Rev. 07 (10), 8038–8047. 10.47191/ijcsrr/v7-i10-64

[B169] Murugesan D. Ray P. C. Bayliss T. Prosser G. A. Harrison J. R. Green K. (2018). 2-mercapto-quinazolinones as inhibitors of type ii nadh dehydrogenase and mycobacterium tuberculosis: structure–activity relationships, mechanism of action and absorption, distribution, metabolism, and excretion characterization. ACS Infect. Dis. 4 (6), 954–969. 10.1021/acsinfecdis.7b00275 29522317 PMC5996347

[B170] Neumann W. Sassone‐Corsi M. Raffatellu M. Nolan E. (2018). Esterase-catalyzed siderophore hydrolysis activates an enterobactin–ciprofloxacin conjugate and confers targeted antibacterial activity. J. Am. Chem. Soc. 140 (15), 5193–5201. 10.1021/jacs.8b01042 29578687 PMC5921047

[B171] Ngambenjawong C. Chan L. Fleming H. Bhatia S. (2022). Conditional antimicrobial peptide therapeutics. Acs Nano 16 (10), 15779–15791. 10.1021/acsnano.2c04162 35980829 PMC9619929

[B172] Niles A. L. Kupcho K. R. Duellman S. Vidugirienė J. Lazar D. Cali J. J. (2018). Abstract 3901: characterizing antibody-drug conjugate cytotoxicity using four different real-time assays. Cancer Res. 78 (13_Suppl. ment), 3901. 10.1158/1538-7445.am2018-3901

[B173] Noy J. Lu H. Hogg P. J. Yang J. Stenzel M. H. (2018). Direct polymerization of the arsenic drug PENAO to obtain nanoparticles with high thiol-reactivity and anti-cancer efficiency. Bioconjugate Chem. 29 (2), 546–558. 10.1021/acs.bioconjchem.8b00032 29346731

[B174] Nriagu J. O. Skaar E. P. Trace metals and infectious diseases (Cambridge, MA: MIT Press Scholarship Online). 10.7551/mitpress/9780262029193.001.0001 33886171

[B175] Omar I. M. Alharas M. M. A. Feizi‐Dehnayebi M. Alharbi S. K. Abo‐Dief H. M. Qasem H. A. (2025). Design, synthesis, physico‐chemical characterization, stability determination, and biomedical applications of some novel tetra‐dentate imine metal chelates supported by theoretical approaches: bridging coordination chemistry and life sciences. Appl. Organomet. Chem. 39 (3), e70056. 10.1002/aoc.70056

[B176] Ou J. Si Y. Goh K. Yasui N. Guo Y. Song J. (2018). Bioprocess development of antibody-drug conjugate production for cancer treatment. Plos One 13 (10), e0206246. 10.1371/journal.pone.0206246 30352095 PMC6198984

[B177] O’Leary M. K. Ahmed A. Alabi C. A. (2023). Development of host-cleavable antibody–bactericide conjugates against extracellular pathogens. ACS Infect. Dis. 9 (2), 322–329. 10.1021/acsinfecdis.2c00492 36626184

[B178] Pal S. Keerthiga G. Eswaran S. (2018). Chemical crosslinking‐mass spectrometry (cxl‐ms) for proteomics, antibody‐drug conjugates (adcs) and cryo‐electron microscopy (cryo‐em). IUBMB Life 70 (10), 947–960. 10.1002/iub.1916 30176115

[B179] Palacio-Castañeda V. Brock R. Verdurmen W. P. R. (2022). “Generation of protein-phosphorodiamidate morpholino oligomer conjugates for efficient cellular delivery via anthrax protective antigen,” in Antisense RNA design, delivery, and analysis. Editors. V. Arechavala-Gomeza, A. Garanto. New York, NY: Methods in Molecular Biology. 2434, 129–141. 10.1007/978-1-0716-2010-6_8 PMC9703282 35213014

[B180] Pan D. Tang Y. Tong J. Xie C. Chen J. Feng C. (2019). An antibody drug conjugate targeting a gsta glycosite-signature epitope of mucin1 expressed by non-small cell lung cancer. 10.1101/2019.12.22.885566 PMC7774737 33084221

[B181] Parakh S. Nicolazzo J. A. Scott A. M. Gan H. (2021). Antibody drug conjugates in glioblastoma – is there a future for them? Front. Oncol. 11, 718590. 10.3389/fonc.2021.718590 34926242 PMC8678283

[B182] Pardridge W. (2020). Treatment of alzheimer’s disease and blood–brain barrier drug delivery. Pharmaceuticals 13 (11), 394. 10.3390/ph13110394 33207605 PMC7697739

[B183] Pardridge W. M. (2023). Treatment of Parkinson’s disease with biologics that penetrate the blood–brain barrier *via* receptor-mediated transport. Front. Aging Neurosci. 15, 1276376. 10.3389/fnagi.2023.1276376 38035276 PMC10682952

[B184] Paull M. L. Johnston T. Ibsen K. N. Bozekowski J. Daugherty P. S. (2019). A general approach for identifying protein epitopes targeted by antibody repertoires using whole proteomes. 10.1101/641787 PMC6730857 31490930

[B185] Peck M. Rothenberg M. Deng R. Lewin‐Koh N. She G. Kamath A. (2019). A phase 1, randomized, single-ascending-dose study to investigate the safety, tolerability, and pharmacokinetics of dsta4637s, an anti-staphylococcus aureus thiomab antibody-antibiotic conjugate, in healthy volunteers. Antimicrob. Agents Chemother. 63 (6). 10.1128/aac.02588-18 30910894 PMC6535527

[B186] Pericolini E. (2018). 4/Epitope unmasking in vulvovaginal candidiasis is associated with hyphal growth and neutrophilic infiltration. 10.26226/morressier.5ac39997d462b8028d89a122 PMC6067721 30063729

[B187] Petersen M. E. Brant M. G. Lasalle M. Das S. Duan R. Wong J. (2024). Design and evaluation of ZD06519, a novel camptothecin payload for antibody drug conjugates. Mol. Cancer Ther. 23 (5), 606–618. 10.1158/1535-7163.mct-23-0822 38354417 PMC11063767

[B188] Peukert C. Vetter A. C. Fuchs H. L. S. Harmrolfs K. Karge B. Stadler M. (2023). Siderophore conjugation with cleavable linkers boosts the potency of rna polymerase inhibitors against multidrug-resistante. coli. Chem. Sci. 14 (20), 5490–5502. 10.1039/d2sc06850h 37234900 PMC10208051

[B189] Piersimoni L. Kastritis P. L. Arlt C. Sinz A. (2021). Cross-linking mass spectrometry for investigating protein conformations and protein–protein interactions─a method for all seasons. Chem. Rev. 122 (8), 7500–7531. 10.1021/acs.chemrev.1c00786 34797068

[B190] Pilarczyk M. Kouril M. Shamsaei B. Vasiliauskas J. Niu W. Mahi N. A. (2019). Connecting omics signatures of diseases, drugs, and mechanisms of actions with iLINCS. 10.1101/826271 PMC9362980 35945222

[B191] Pincus S. H. Luo K. Peters T. Gordy J. T. Cole F. M. Klug G. (2025). J3exoa: a novel anti-hiv immunotoxin fusion of anti-gp120 j3vhh and pe38 fragment of pseudomonas exotoxin a. Pharmaceuticals 18 (9), 1305. 10.3390/ph18091305 41011175 PMC12472382

[B192] Pires D. Valente S. Calado M. Mandal M. Azevedo‐Pereira J. M. Anes E. (2021). Repurposing saquinavir for host-directed therapy to control Mycobacterium tuberculosis infection. Front. Immunol. 12, 647728. 10.3389/fimmu.2021.647728 33841429 PMC8032898

[B193] Pishesha N. Harmand T. Smeding L. Ma W. Ludwig L. Janssen R. (2021). Induction of antigen-specific tolerance by nanobody–antigen adducts that target class-ii major histocompatibility complexes. Nat. Biomed. Eng. 5 (11), 1389–1401. 10.1038/s41551-021-00738-5 34127819

[B195] Qian L. Lin X. Gao X. Khan R. U. Liao J. Du S. (2023). The dawn of a new era: targeting the “Undruggables” with antibody-based therapeutics. Chem. Rev. 123 (12), 7782–7853. 10.1021/acs.chemrev.2c00915 37186942

[B196] Qin L. Hu N. Zhang Y. Yang J. Zhao L. Zhang X. (2023). Antibody-antibiotic conjugate targeted therapy for orthopedic implant-associated intracellular s. Aureus Infections. J. Adv. Res. 65, 239–255. 10.1016/j.jare.2023.12.001 38048846 PMC11519013

[B197] Quinn H. M. Emerson K. Shukait S. Czeryba N. Kucharczyk L. Zaitouna A. (2025). Abstract b089: development, characterization, and humanization of nci-n87 human gastric carcinoma xenograft model in nsg mice. Mol. Cancer Ther. 24 (10_Suppl. ment), B089. 10.1158/1535-7163.targ-25-b089

[B199] Rao P. G. Lambert G. S. Upadhyay C. (2023). Broadly neutralizing antibody epitopes on hiv-1 particles are exposed after virus interaction with host cells. J. Virology 97 (9), e0071023. 10.1128/jvi.00710-23 37681958 PMC10537810

[B200] Ras-Carmona A. Pelaez-Prestel H. F. Lafuente E. M. Reche P. A. (2021). Bceps: a web server to predict linear b cell epitopes with enhanced immunogenicity and cross-reactivity. Cells 10 (10), 2744. 10.3390/cells10102744 34685724 PMC8534968

[B201] Riaz A. Fatima Z. Mansoor M. Khalid A. Chaudhary A. Javaid Z. (2025). Cell-penetrating peptides: enhancing antimicrobial efficacy against intracellular pathogens. Int. J. Sci. Res. Archive 14 (2), 1754–1766. 10.30574/ijsra.2025.14.2.0538

[B202] Riccardi F. Bo M. D. Macor P. Toffoli G. (2023). A comprehensive overview on antibody-drug conjugates: from the conceptualization to cancer therapy. Front. Pharmacol. 14. 10.3389/fphar.2023.1274088 PMC10544916 37790810

[B203] Richardson N. C. Kasamon Y. L. Chen H. Claro R. A. Ye J. Blumenthal G. M. (2019). FDA approval summary: Brentuximab vedotin in first-line treatment of peripheral T-Cell lymphoma. Oncol. 24 (5), e180–e187. 10.1634/theoncologist.2019-0098 30914464 PMC6516120

[B204] Rodríguez‐Martínez M. Boissière T. González M. N. Litchfield K. Mitter R. Walker J. (2020). Evidence that STK19 is not an NRAS-dependent Melanoma driver. Cell 181 (6), 1395–1405.e11. 10.1016/j.cell.2020.04.014 32531245 PMC7298618

[B205] Rossin R. Robillard M. S. (2021). Click-cleavable ADC linkers. Chem. Linkers Antibody–Drug Conjugates (ADCs), 263–285. 10.1039/9781839165153-00263

[B206] Ruddle B. T. Fleming R. Wu H. Gao C. Dimasi N. (2019). Characterization of disulfide bond rebridged fab–drug conjugates prepared using a dual maleimide pyrrolobenzodiazepine cytotoxic payload. ChemMedChem 14 (12), 1185–1195. 10.1002/cmdc.201900077 30980702

[B207] Rudin C. M. Reck M. Johnson M. L. Blackhall F. Hann C. L. Yang J. C. (2023). Emerging therapies targeting the delta-like ligand 3 (DLL3) in small cell lung cancer. J. Hematol. Oncol. 16 (1), 66. 10.1186/s13045-023-01464-y 37355629 PMC10290806

[B208] Ruma Y. N. Nannenga B. L. Gonen T. (2025). Unraveling atomic complexity from frozen samples. Struct. Dyn. 12 (2), 020901. 10.1063/4.0000303 40255534 PMC12009148

[B210] Saeed U. Insaf R. A. Piracha Z. Z. Tariq M. N. Sohail A. Abbasi U. A. (2023). Crisis averted: a world united against the menace of multiple drug-resistant superbugs -pioneering anti-amr vaccines, rna interference, nanomedicine, crispr-based antimicrobials, bacteriophage therapies, and clinical artificial intelligence strategies to safeguard global antimicrobial arsenal. Front. Microbiol. 14, 1270018. 10.3389/fmicb.2023.1270018 38098671 PMC10720626

[B211] Sasso J. M. Tenchov R. Bird R. E. Iyer K. A. Ralhan K. Rodríguez Y. (2023). The evolving landscape of antibody–drug conjugates: in depth analysis of recent research progress. Bioconjugate Chem. 34 (11), 1951–2000. 10.1021/acs.bioconjchem.3c00374 37821099 PMC10655051

[B212] Savoy E. A. Olatunji F. P. Yoon H. Mesbahi N. Knight J. R. Berkman C. E. (2021). Acid-labile linkers. Chem. Linkers Antibody–Drug Conjugates (ADCs), 213–231. 10.1039/9781839165153-00213

[B213] Schmitt S. Machui P. Mai I. Herterich S. Wunder S. Cyprys P. (2023). Design and evaluation of phosphonamidate-linked exatecan constructs for highly loaded, stable, and efficacious antibody–drug conjugates. Mol. Cancer Ther. 23 (2), 199–211. 10.1158/1535-7163.mct-23-0359 37828728 PMC10831470

[B214] Schoehn G. Chenavier F. Crépin T. (2023). Advances in structural virology *via* cryo-em in 2022. Viruses 15 (6), 1315. 10.3390/v15061315 37376615 PMC10304789

[B215] Schwach J. Abdellatif M. Stengl A. (2022). More than toxins—current prospects in designing the next generation of antibody drug conjugates. Front. Bioscience-Landmark 27 (8), 240. 10.31083/j.fbl2708240 36042167

[B216] Šeborová K. Koucká K. Spálenková A. Holý P. Ehrlichová M. Sychra T. (2022). Anticancer regimens containing third generation taxanes SB-T-121605 and SB-T-121606 are highly effective in resistant ovarian carcinoma model. Front. Pharmacol. 13, 971905. 10.3389/fphar.2022.971905 36438837 PMC9681785

[B217] Sen S. Xavier J. Kumar N. Ahmad M. Z. Ranjan O. P. (2023). Exosomes as natural nanocarrier-based drug delivery system: recent insights and future perspectives. 3 Biotech. 13 (3), 101. 10.1007/s13205-023-03521-2 36860361 PMC9970142

[B218] Shekhar S. Sharma S. Okolie J. A. Kumar A. Sharma B. Meena M. K. (2022). Synthesis, structural elucidation, biological screening, and density functional theory calculations of Cu(II), Ni(II), Mn(II), and Co(II) complexes of 20 Z‐N‐((Z)‐2‐(6‐nitrobenzo[d]thiazol‐2‐ylimino)‐1,2‐diphenylethylidene)‐5‐nitrobenzo[d]thiazol‐2‐amine schiff base ligand. Appl. Organomet. Chem. 36 (8), e6766. 10.1002/aoc.6766

[B219] Shi R. Jia L. Lv Z. Cui J. (2025). Another power of antibody-drug conjugates: immunomodulatory effect and clinical applications. Front. Immunol. 16, 1632705. 10.3389/fimmu.2025.1632705 40909270 PMC12404974

[B220] Shia C. Wen S. Hsu R. Tu J. Chang H. Weng H. (2025). Preclinical pharmacokinetic, pharmacodynamic, and safety profile of OBI-992: a novel TROP2-Targeted antibody–drug conjugate. Mol. Cancer Ther. OF1-OF10 24, 1938–1947. 10.1158/1535-7163.mct-24-1176 40762235 PMC12670072

[B221] Shih C. Lin Y. Luo H. Sung W. (2024). Antibody-drug conjugates targeting her2 for the treatment of urothelial carcinoma: potential therapies for her2-positive urothelial carcinoma. Front. Pharmacol. 15, 1326296. 10.3389/fphar.2024.1326296 38572425 PMC10987710

[B222] Shim H. (2020). Bispecific antibodies and antibody–drug conjugates for cancer therapy: technological considerations. Biomolecules 10 (3), 360. 10.3390/biom10030360 32111076 PMC7175114

[B223] Shivatare V. S. Huang H. Tseng T. Chuang P. Zeng Y. Wong C. (2023). Probing the internalization and efficacy of antibody‐drug conjugate via site‐specific Fc‐Glycan labelling of a homogeneous antibody targeting SSEA‐4 bearing tumors. Israel J. Chem. 63 (10-11), e202300042. 10.1002/ijch.202300042 38348405 PMC10861153

[B224] Silva E. C. D. D’Ancona C. A. L. Justiniano H. Calco V. Sensoy D. Villa P. (2024). New therapeutic combination to enhance endocytosis of antibodies and nucleic-acid aptamers targeting EGFR in glioblastoma cells. 10.1101/2024.10.22.617611 42390764

[B225] Singh A. P. Guo L. Verma A. Wong G. Shah D. K. (2019). A cell-level systems pk-pd model to characterize *in vivo* efficacy of adcs. Pharmaceutics 11 (2), 98. 10.3390/pharmaceutics11020098 30823607 PMC6409735

[B226] Sograte‐Idrissi S. Oleksiievets N. Isbaner S. Eggert-Martinez M. Enderlein J. Tsukanov R. (2019). Nanobody detection of standard fluorescent proteins enables multi-target DNA-PAINT with high resolution and minimal displacement errors. Cells 8 (1), 48. 10.3390/cells8010048 30646582 PMC6357156

[B227] Sovari S. N. Zobi F. (2020). Recent studies on the antimicrobial activity of transition metal complexes of groups 6–12. Chemistry 2 (2), 418–452. 10.3390/chemistry2020026

[B228] Spangler B. Kline T. Hanson J. Li X. Zhou S. Wells J. A. (2018). Toward a ferrous iron-cleavable linker for antibody–drug conjugates. Mol. Pharm. 15 (5), 2054–2059. 10.1021/acs.molpharmaceut.8b00242 29569925

[B229] Spring L. M. Wu B. Liu T. Geisberg J. Cristea S. Bossuyt V. (2024). Abstract PR08: intratumoral heterogeneity drives resistance to antibody drug conjugate therapy: analysis of the NeoSTAR trial of neoadjuvant sacituzumab govitecan for localized TNBC. Cancer Res. 84 (3_Suppl. ment_1), PR08. 10.1158/1538-7445.advbc23-pr08

[B231] Stoessel A. Groysbeck N. Guyot L. Barret L. Nominé Y. Nguekeu-Zebaze L. (2020). Modular conjugation of a potent Anti-HER2 immunotoxin using coassociating peptides. Bioconjugate Chem. 31 (10), 2421–2430. 10.1021/acs.bioconjchem.0c00482 32996763

[B232] Stoppa I. Dianzani C. Clemente N. Bozza A. Bordano V. Garelli S. (2024). Alendronate-grafted nanoemulsions for bone-targeted vincristine delivery: preliminary studies on cell and animal models. Biomolecules 14 (2), 238. 10.3390/biom14020238 38397475 PMC10886946

[B233] Su D. Zhang D. (2021). Linker design impacts antibody-drug conjugate pharmacokinetics and efficacy *via* modulating the stability and payload release efficiency. Front. Pharmacol. 12, 687926. 10.3389/fphar.2021.687926 34248637 PMC8262647

[B234] Su F. Srinivasan S. Lee B. Chen J. Convertine A. J. West T. E. (2018). Macrophage-targeted drugamers with enzyme-cleavable linkers deliver high intracellular drug dosing and sustained drug pharmacokinetics against alveolar pulmonary infections. J. Control. Release 287, 1–11. 10.1016/j.jconrel.2018.08.014 30099019 PMC6223132

[B235] Su Z. Xiao D. Xie F. Liu L. Wang Y. Fan S. (2021). Antibody–drug conjugates: recent advances in linker chemistry. Acta Pharm. Sin. B 11 (12), 3889–3907. 10.1016/j.apsb.2021.03.042 35024314 PMC8727783

[B236] Sun W. Weng W. Shi J. Ma B. DeMarco K. D. Gui F. (2025). Sparc: a multipayload adc architecture for programmable drug combinations. Bioconjugate Chem. 36 (10), 2158–2171. 10.1021/acs.bioconjchem.5c00239 40958380 PMC12532090

[B237] Suzuki Y. Zhou S. Ota Y. Harrington M. Miyagi E. Takagi H. (2023). Toxicity profiles of antibody-drug conjugates for anticancer treatment: a systematic review and meta-analysis. JNCI Cancer Spectr. 7 (5), pkad069. 10.1093/jncics/pkad069 37756687 PMC10579782

[B238] Swain A. Gnanasekar P. Prava J. Rajeev A. C. Kesarwani P. Lahiri C. (2021). A comparative genomics approach for shortlisting broad-spectrum drug targets in nontuberculous mycobacteria. Microb. Drug Resist. 27 (2), 212–226. 10.1089/mdr.2020.0161 32936741

[B239] Taiariol L. Chaix C. Farre C. Moreau E. (2021). Click and bioorthogonal chemistry: the future of active targeting of nanoparticles for nanomedicines? Chem. Rev. 122 (1), 340–384. 10.1021/acs.chemrev.1c00484 34705429

[B240] Tanaka Y. Tanaka M. Miyazawa H. Terashima R. Miyazawa M. Ikuma M. (2024). Postmarket safety communications on drugs approved in Japan: a 25‐year analysis. Clin. Transl. Sci. 17 (4), e13803. 10.1111/cts.13803 38651283 PMC11036129

[B241] Tarasova O. Ivanov S. M. Filimonov D. Poroikov V. (2020). Data and text mining help identify key proteins involved in the molecular mechanisms shared by SARS-CoV-2 and HIV-1. Molecules 25 (12), 2944. 10.3390/molecules25122944 32604797 PMC7357070

[B242] Tas J. M. J. Koo J. Lin Y. Xie Z. Steichen J. M. Jackson A. M. (2022). Antibodies from primary humoral responses modulate the recruitment of naive b cells during secondary responses. Immunity 55 (10), 1856–1871.e6. 10.1016/j.immuni.2022.07.020 35987201 PMC9350677

[B243] Tashima T. (2022). Delivery of drugs into cancer cells using antibody–drug conjugates based on receptor-mediated endocytosis and the enhanced permeability and retention effect. Antibodies 11 (4), 78. 10.3390/antib11040078 36546903 PMC9774242

[B244] Tateishi M. Goto K. Hishinuma E. Matsukawa N. Kishimoto T. Tanaka K. (2025). Double prenylation of budding yeast Ykt6 regulates cell wall integrity and autophagy. J. Biol. Chem. 301 (4), 108384. 10.1016/j.jbc.2025.108384 40049413 PMC12001115

[B245] Tatiparti K. Rauf M. Sau S. Iyer A. K. (2020). Carbonic Anhydrase-IX guided albumin nanoparticles for hypoxia-mediated triple-negative breast cancer cell killing and imaging of patient-derived tumor. Molecules 25 (10), 2362. 10.3390/molecules25102362 32438691 PMC7287925

[B246] Theocharopoulos C. Lialios P. Gogas H. Ziogas D. C. (2020). An overview of antibody–drug conjugates in oncological practice. Therm. Adv. Med. Oncol. 12, 1758835920962997. 10.1177/1758835920962997 33088347 PMC7543133

[B247] Theocharopoulos C. Lialios P. Samarkos M. Gogas H. Ziogas D. C. (2021). Antibody-drug conjugates: functional principles and applications in oncology and beyond. Vaccines 9 (10), 1111. 10.3390/vaccines9101111 34696218 PMC8538104

[B248] Tolosa E. J. Yang L. Ayers-Ringler J. Suzuki S. Mallareddy J. R. Schaefer-Klein J. (2024). Proteolysis targeting chimera (protac)-driven antibody internalization of oncogenic cell surface receptors. Commun. Biol. 7 (1), 1719. 10.1038/s42003-024-07439-0 39741170 PMC11688428

[B249] Tonon G. Rizzolio F. Visentin F. Scattolin T. (2024). Antibody drug conjugates for cancer therapy: from metallodrugs to nature-inspired payloads. Int. J. Mol. Sci. 25 (16), 8651. 10.3390/ijms25168651 39201338 PMC11355040

[B250] Torres J. N. Lara T. Grijalva M. (2022). Nanoparticle applications in intracellular infections. J. Drug Deliv. Ther. 12 (5-S), 217–219. 10.22270/jddt.v12i5-s.5738

[B251] Trail A. Rogers J. E. Ajani J. A. (2023). Can you establish the cause of this patient’s shortness of breath? J. Adv. Pract. Oncol. 14 (5), 440–443. 10.6004/jadpro.2023.14.5.8 37576362 PMC10414527

[B252] Travassos M. A. Niangaly A. Bailey J. A. Ouattara A. Coulibaly D. Lyke K. E. (2018). Children with cerebral malaria or severe malarial anaemia lack immunity to distinct variant surface antigen subsets. Sci. Rep. 8 (1), 6281. 10.1038/s41598-018-24462-4 29674705 PMC5908851

[B253] Tsuchikama K. Anami Y. Ha S. Y. Yamazaki C. M. (2024). Exploring the next generation of antibody–drug conjugates. Nat. Rev. Clin. Oncol. 21 (3), 203–223. 10.1038/s41571-023-00850-2 38191923

[B254] Turner H. L. Pallesen J. Lang S. Bangaru S. Urata S. Li S. (2018). Potent anti-influenza h7 human monoclonal antibody induces separation of hemagglutinin receptor binding head domains. 10.1101/436642 PMC6375650 30716060

[B255] Tvilum A. Johansen M. Glud L. Ivarsen D. Khamas A. Carmali S. (2023). Antibody‐drug conjugates to treat bacterial biofilms *via* targeting and extracellular drug release. Adv. Sci. 10 (23), e2301340. 10.1002/advs.202301340 37290045 PMC10427384

[B256] Umezaki Y. Matsumoto K. Ikawa K. Yokoyama Y. Enoki Y. Shigemi A. (2022). Concentration-dependent activity of pazufloxacin against pseudomonas aeruginosa: an *in vivo* pharmacokinetic/pharmacodynamic study. Antibiotics 11 (7), 982. 10.3390/antibiotics11070982 35884236 PMC9312304

[B257] Usama S. M. Marker S. C. Caldwell D. R. Patel N. L. Feng Y. Kalen J. D. (2021). Targeted fluorogenic cyanine carbamates enable *in vivo* analysis of antibody–drug conjugate linker chemistry. J. Am. Chem. Soc. 143 (51), 21667–21675. 10.1021/jacs.1c10482 34928588 PMC10263178

[B258] Usuda K. Iwai S. Yamagata A. Iijima Y. Motono N. Matoba M. (2021). Whole-lesion apparent diffusion coefficient histogram analysis: significance for discriminating lung cancer from pulmonary abscess and mycobacterial infection. Cancers 13 (11), 2720. 10.3390/cancers13112720 34072867 PMC8198705

[B259] Vafadar A. Taheri‐Anganeh M. Movahedpour A. Jamali Z. Irajie C. Ghasemi Y. (2020). *In silico* design and evaluation of scFv-CdtB as a novel immunotoxin for breast cancer treatment. Int. J. Cancer Manag. 13 (1). 10.5812/ijcm.96094

[B260] Volpedo G. Costa L. Ryan N. Halsey G. Satoskar A. Oghumu S. (2019). Nanoparticulate drug delivery systems for the treatment of neglected tropical protozoan diseases. J. Venom. Animals Toxins Incl. Trop. Dis. 25, e144118. 10.1590/1678-9199-jvatitd-1441-18 31130996 PMC6483407

[B261] Walker J. A. Sorkin M. Ledesma F. Kabaria S. R. Barfield R. M. Rabuka D. (2019). Hydrophilic sequence-defined cross-linkers for antibody–drug conjugates. Bioconjugate Chem. 30 (11), 2982–2988. 10.1021/acs.bioconjchem.9b00713 31671265

[B262] Walter J. D. Scherer M. Hutter C. A. J. Garaeva A. A. Zimmermann I. Wyss M. (2022). Biparatopic sybodies neutralize sars‐cov‐2 variants of concern and mitigate drug resistance. EMBO Rep. 23 (4), e54199. 10.15252/embr.202154199 35253970 PMC8982573

[B263] Wang Y. Fan S. Xiao D. Xie F. Li W. Zhong W. (2019). Novel silyl ether-based acid-cleavable Antibody-MMAE conjugates with appropriate stability and efficacy. Cancers 11 (7), 957. 10.3390/cancers11070957 31288450 PMC6678733

[B264] Wang Y. Liu L. Fan S. Xiao D. Xie F. Li W. (2020). Antibody-drug conjugate using ionized Cys-Linker-MMAE as the potent payload shows optimal therapeutic safety. Cancers 12 (3), 744. 10.3390/cancers12030744 32245171 PMC7140114

[B265] Wang Y. Jian M. Chen P. Tsou J. Truong L. P. Wang Y. (2021). Ferritin conjugates with multiple clickable amino acids encoded by C-Terminal engineered Pyrrolysyl-tRNA synthetase. Front. Chem. 9, 779976. 10.3389/fchem.2021.779976 34900939 PMC8655692

[B266] Wang X. Xu W. Liu Z. Wu Y. Wang Q. Cao M. (2022). A toxin-conjugated recombinant protein targeting gp120 and gp41 for inactivating HIV-1 virions and killing latency-reversing agent-reactivated latent cells. mBio 13 (1), e0338421. 10.1128/mbio.03384-21 35038908 PMC8764533

[B267] Wang K. Neumann C. Epp A. Zeng W. Griffith T. S. Ferguson D. M. (2023). Abstract 1542: generation of an antibody-drug conjugate-optimized TLR 7/8 agonist payload. Cancer Res. 83 (7_Suppl. ment), 1542. 10.1158/1538-7445.am2023-1542

[B268] Wang J. Xiong Z. Fan Y. Wang H. An C. Wang B. (2024). Lignin/surfactin coacervate as an eco-friendly pesticide carrier and antifungal agent against phytopathogen. ACS Nano 18 (33), 22415–22430. 10.1021/acsnano.4c07173 39126678

[B269] Wang Y. Lv H. Teo Q. W. Lei R. Gopal A. B. Ouyang W. O. (2024). An explainable language model for antibody specificity prediction using curated influenza hemagglutinin antibodies. Immunity 57 (10), 2453–2465.e7. 10.1016/j.immuni.2024.07.022 39163866 PMC11464180

[B270] Wang K. Yang R. Li J. Wang H. Li W. He J. (2025a). Nanocarrier-based targeted drug delivery for alzheimer’s disease: addressing neuroinflammation and enhancing clinical translation. Front. Pharmacol. 16, 1591438. 10.3389/fphar.2025.1591438 40438598 PMC12116324

[B271] Wang K. Wang C. Yang H. Chen G. Wang K. Ji P. (2025b). A dual-targeting peptide–drug conjugate based on CXCR4 and FOLR1 inhibits triple-negative breast cancer. Acta Pharm. Sin. B 15 (10), 4995–5009. 10.1016/j.apsb.2025.06.012 41132836 PMC12541585

[B272] Wang S. Yan X. Yang C. Naranmandura H. (2022). The landscape of nucleic-acid-based aptamers for treatment of hematologic malignancies: challenges and future directions. Bioengineering 9 (11), 635. 10.3390/bioengineering9110635 36354547 PMC9687288

[B273] Watanabe T. Fujii T. Matsuda Y. (2024). Exo-cleavable linkers: enhancing stability and therapeutic efficacy in antibody-drug conjugates. J. Synthetic Org. Chem. Jpn. 82 (11), 1117–1124. 10.5059/yukigoseikyokaishi.82.1117 PMC11513888 39410752

[B274] Wehrmüller J. Frei J. Hechler T. Kulke M. Pahl A. Béhé M. (2024). Site‐specific modification of native IgGs with flexible drug‐load. ChemBioChem 26 (8), e202400511. 10.1002/cbic.202400511 39305147 PMC12007069

[B275] Wintjens R. Bifani A. M. Bifani P. (2020). Impact of glycan cloud on the b-cell epitope prediction of sars-cov-2 spike protein. NPJ Vaccines 5 (1), 81. 10.1038/s41541-020-00237-9 32944295 PMC7474083

[B276] Wu X. Jin S. Ding C. Wang Y. He D. Liu Y. (2022). Mesenchymal stem cell-derived exosome therapy of microbial diseases: from bench to bed. Front. Microbiol. 12, 804813. 10.3389/fmicb.2021.804813 35046923 PMC8761948

[B277] Xu J. Li X. Du Y. (2022). Antibody–pattern recognition receptor agonist conjugates: a promising therapeutic strategy for cancer. Adv. Biol. 6 (3), e2101065. 10.1002/adbi.202210065 35122418

[B278] Yamazaki C. M. Yamaguchi A. Anami Y. Xiong W. Otani Y. Lee J. (2021). Antibody-drug conjugates with dual payloads for combating breast tumor heterogeneity and drug resistance. Nat. Commun. 12 (1), 3528. 10.1038/s41467-021-23793-7 34112795 PMC8192907

[B279] Yamazaki S. Ito K. Aoki T. Arashida N. Watanabe T. Fujii T. (2024). Biological evaluation of antibody-drug conjugates produced by tag-free lipoate ligase a modification. Biochemistry 63 (5), 644–650. 10.1021/acs.biochem.3c00513 38350078

[B280] Yamazoe S. Cheng Q. Kotapati S. Rangan V. S. Sung M. Deshpande M. (2025). The impact of conjugation mode and site on tubulysin antibody‐drug‐conjugate efficacy and stability. ChemistryOpen 14 (8), e202400522. 10.1002/open.202400522 40434212 PMC12368874

[B281] Yang X. Seol H. Lin W. Xu X. Shen B. Qiu H. (2021). Site-specific quantitation of drug conjugations on antibody–drug conjugates (adcs) using a protease-assisted drug deconjugation and linker-like labeling (paddll) method. Anal. Chem. 93 (27), 9549–9558. 10.1021/acs.analchem.1c01619 34196532

[B282] Yang F. Wang L. Tian X. Qiu X. Chen J. Qi W. (2024). Abstract LB425: a tumor microenvironment-targeting CD98-directed ADC confers robust anti-tumor activity in multiple cancers with favorable pharmacokinetics and safety profiles in preclinical models. Cancer Res. 84 (7_Suppl. ment), LB425. 10.1158/1538-7445.am2024-lb425

[B283] Ye M. Zhao Y. Wang Y. Yodsanit N. Xie R. Gong S. (2020). Ph‐responsive polymer–drug conjugate: an effective strategy to combat the antimicrobial resistance. Adv. Funct. Mater. 30 (39), 2002655. 10.1002/adfm.202002655

[B284] Yelamali A. Chendamarai E. Ritchey J. Rettig M. DiPersio J. Persaud S. (2024). Streptavidin-drug conjugates streamline optimization of antibody-based conditioning for hematopoietic stem cell transplantation. bioRxiv., 2024.02.12.579199. 10.1101/2024.02.12.579199 41574174 PMC12823147

[B285] Yeo H. Won J. Gwon L. Roh S. Lee W. Lim K. (2024). CdSe quantum dot-based delivery system for CRISPR-Cas9 mediated microglial gene modulation. ACS Appl. Nano Mater. 7 (20), 24013–24027. 10.1021/acsanm.4c04586

[B286] Yoder N. C. Bai C. Tavares D. Widdison W. C. Whiteman K. R. Wilhelm A. (2019). A case study comparing heterogeneous Lysine- and site-specific cysteine-conjugated maytansinoid antibody-drug conjugates (ADCs) illustrates the benefits of lysine conjugation. Mol. Pharm. 16 (9), 3926–3937. 10.1021/acs.molpharmaceut.9b00529 31287952

[B287] Yu L. Shang Z. Jin Q. Chan S. Y. Hong W. Li N. (2022). Antibody–antimicrobial conjugates for combating antibiotic resistance. Adv. Healthc. Mater. 12 (1), e2202207. 10.1002/adhm.202202207 36300640

[B288] Yuan H. Zhao H. Peng K. Qi R. Bai H. Zhang P. (2019). Conjugated polymer-quantum dot hybrid materials for pathogen discrimination and disinfection. ACS Appl. Mater. Interfaces 12 (19), 21263–21269. 10.1021/acsami.9b17783 31825194

[B289] Zabara M. Ren Q. Amenitsch H. Salentinig S. (2021). Bioinspired antimicrobial coatings from peptide-functionalized liquid crystalline nanostructures. ACS Appl. Bio Mater. 4 (6), 5295–5303. 10.1021/acsabm.1c00415 35007010

[B290] Zaleski M. Chase L. Hood E. Wang Z. Nong J. Espy C. (2024). Conjugation chemistry markedly impacts toxicity and biodistribution of targeted nanoparticles, mediated by complement activation. Adv. Mater. 37 (5), 2409945. 10.1002/adma.202409945 39663706 PMC11795710

[B291] Zelter T. Strahilevitz J. Simantov K. Yajuk O. Adams Y. Jensen A. T. R. (2022). Neutrophils impose strong immune pressure against pfemp1 variants implicated in cerebral malaria. EMBO Rep. 23 (6), e53641. 10.15252/embr.202153641 35417070 PMC9171683

[B292] Zhai L. Zhang L. Jiang Y. Li B. Yang M. Khrustalev V. V. (2022). Broadly neutralizing antibodies recognizing different antigenic epitopes act synergistically against the influenza b virus. J. Med. Virology 95 (1), e28106. 10.1002/jmv.28106 36039848

[B293] Zhang Y. (2025). Advances in the application of click chemistry in the research of targeted therapeutic drugs. Transactions on materials. Biotechnol. Life Sci. 8, 377–382. 10.62051/y0yezt68

[B294] Zhang D. Yu S. Khojasteh S. C. Ma Y. Pillow T. H. Sadowsky J. (2018). Intratumoral payload concentration correlates with the activity of antibody–drug conjugates. Mol. Cancer Ther. 17 (3), 677–685. 10.1158/1535-7163.mct-17-0697 29348271

[B295] Zhang Y. Ding M. Wang L. Yin S. Zhang L. Cao X. (2023). Synthesis and biological evaluation of novel Quaternary ammonium antibody drug conjugates based on camptothecin derivatives. Plos One 18 (12), e0292871. 10.1371/journal.pone.0292871 38113206 PMC10729962

[B296] Zhang B. Wang M. Sun L. Liu J. Yin L. Xia M. (2024). Recent advances in targeted cancer therapy: are pdcs the next generation of adcs? J. Med. Chem. 67 (14), 11469–11487. 10.1021/acs.jmedchem.4c00106 38980167

[B297] Zhang Y. Wang L. Cao X. Song R. Yin S. Cheng Z. (2024). Evaluation of double self-immolative linker-based antibody–drug conjugate FDA022-BB05 with enhanced therapeutic potential. J. Med. Chem. 67 (21), 19852–19873. 10.1021/acs.jmedchem.4c02243 39444220

[B298] Zhao B. Chen S. Hong Y. Jia L. Zhou Y. He X. (2022). Research progress of conjugated nanomedicine for cancer treatment. Pharmaceutics 14 (7), 1522. 10.3390/pharmaceutics14071522 35890416 PMC9315807

[B299] Zhu F. Sun H. Lü Q. Fang Y. Xu J. Ju C. (2023). Abstract 3242: efficacy evaluation by novel humanized mouse models for preclinical study of adcs combined with immunotherapy. Cancer Res. 83 (7_Suppl. ment), 3242. 10.1158/1538-7445.am2023-3242

[B300] Zhu K. Yuan C. Du Y. Sun K. Zhang X. Vogel H. (2023). Applications and prospects of cryo-em in drug discovery. Mil. Med. Res. 10 (1), 10. 10.1186/s40779-023-00446-y 36872349 PMC9986049

[B301] Zimmerman B. S. Esteva F. J. (2024). Next-generation her2-targeted antibody–drug conjugates in breast cancer. Cancers 16 (4), 800. 10.3390/cancers16040800 38398191 PMC10887217

[B302] Zong Q. Zhu F. Wu S. Peng L. Mou Y. Miao K. (2020). Advanced pneumonic type of lung adenocarcinoma: survival predictors and treatment efficacy of the tumor. Tumori J. 107 (3), 216–225. 10.1177/0300891620947159 32762285

[B303] Бурмистров В. А. Bogdanchikova N. Gyusan A. O. Ураскулова Б. Б. Almanza‐Reyes H. Alvarado-Vera M. (2021). Prospects for the use of nanostructured silver preparations for the control of infections diseases, including covid-19. Сибирский Научный Медицинский Журнал 41 (5), 4–15. 10.18699/ssmj20210501

